# Genetic Suppression of Basement Membrane Defects in *Caenorhabditis elegans* by Gain of Function in Extracellular Matrix and Cell-Matrix Attachment Genes

**DOI:** 10.1534/genetics.118.300731

**Published:** 2018-02-12

**Authors:** Jennifer R. Gotenstein, Cassidy C. Koo, Tiffany W. Ho, Andrew D. Chisholm

**Affiliations:** Section of Cell and Developmental Biology, Division of Biological Sciences, University of California, San Diego, La Jolla, California 92093

**Keywords:** Peroxidasin, Sulfilimine bond, Myotactin, Spondin, Type IV collagen

## Abstract

Basement membranes are extracellular matrices essential for embryonic development in animals. Peroxidasins are extracellular peroxidases implicated in the unique sulfilimine cross-links between type IV basement membrane collagens. Loss of function in the *Caenorhabditis elegans* peroxidasin PXN-2 results in fully penetrant embryonic or larval lethality. Using genetic suppressor screening, we find that the requirement for PXN-2 in development can be bypassed by gain of function in multiple genes encoding other basement membrane components, or proteins implicated in cell-matrix attachment. We identify multiple alleles of *let-805*, encoding the transmembrane protein myotactin, which suppress phenotypes of *pxn-2* null mutants and of other basement membrane mutants such as F-spondin/*spon-1*. These *let-805* suppressor alleles cause missense alterations in two pairs of FNIII repeats in the extracellular domain; they act dominantly and have no detectable phenotypes alone, suggesting they cause gain of function. We also identify suppressor missense mutations affecting basement membrane components type IV collagen (*emb-9*, *let-2*) and perlecan (*unc-52*), as well as a mutation affecting spectraplakin (*vab-10*), a component of the epidermal cytoskeleton. These suppressor alleles do not bypass the developmental requirement for core structural proteins of the basement membrane such as laminin or type IV collagen. In conclusion, putative gain-of-function alterations in matrix proteins or in cell-matrix receptors can overcome the requirement for certain basement membrane proteins in embryonic development, revealing previously unknown plasticity in the genetic requirements for the extracellular matrix.

BASEMENT membranes (BMs) or basal laminae are specialized extracellular matrices found at the basal surfaces of nearly all animal tissues (Jayadev and Sherwood 2017). BMs are critical for numerous developmental processes, from cell differentiation and specification to organogenesis and tissue growth (Wiradjaja *et al.* 2010; Sherwood 2015). BMs also function in tissue maintenance and provide a barrier for cell invasion as in metastasis of tumor cells. Complete loss of function in BM components often results in embryonic lethality (Smyth *et al.* 1999), whereas partial loss of function in humans results in a variety of genetic disorders, including muscular dystrophy, epidermolysis bullosa (severe skin blistering), ocular defects, and renal disease, as seen in Alport syndrome (Pozzi *et al.* 2017).

Assembly of the first BMs in the early embryo involves a series of partly independent yet coordinated self-assembly processes. Laminin, type IV collagen, heparan sulfate proteoglycans (HSPGs) and nidogen are the most prominent and conserved protein components of BMs (Yurchenco 2011; Clay and Sherwood 2015; Fidler *et al.* 2017). Laminins are required for initial BM assembly, and self-assemble into a cell-associated network independently of type IV collagen, as reviewed in Hohenester and Yurchenco (2013). The laminin network polymerizes *in vivo* through binding to cell surface receptors, including integrin and dystroglycan; laminin can also be anchored to cells through HSPGs or sulfated glycolipids (Hohenester and Yurchenco 2013). Type IV collagen heterotrimers assemble into higher-order networks via lateral associations and covalent cross-links between N-terminal domains (Timpl *et al.* 1981; Yurchenco and Ruben 1987; Chioran *et al.* 2017). Additionally, a peroxidasin-mediated sulfilimine bond links the noncollagenous C-terminal (NC1) domains of type IV collagen (Bhave *et al.* 2012).

In the nematode *Caenorhabditis elegans*, BMs are present between most tissue types and contain the core components such as laminin and type IV collagens. Loss of laminin causes defects in development beginning in late gastrulation (Huang *et al.* 2003). *C. elegans* encodes two type IV collagen α-chains, EMB-9 α1 and LET-2 α2, which form α1α1α2 trimers (Sibley *et al.* 1994; Gupta *et al.* 1997; Kubota *et al.* 2008). Complete loss of type IV collagen function also results in developmental arrest later in embryogenesis; weak hypomorphic alleles of type IV collagen, typically altering a Gly of the Gly-X-Y collagenous motif, result in lower penetrance or temperature-dependent lethality (Gupta *et al.* 1997).

Studies of human epidermal BM support the hypothesis that type IV collagen networks can form layers separate from laminin networks, and have suggested that these layers are connected by the HSPG perlecan (Behrens *et al.* 2012). Perlecan has many roles in development and in other processes (Knox and Whitelock 2006). In *Drosophila*, perlecan localization to BM requires type IV collagen, and perlecan is required to maintain tension of the BM (Pastor-Pareja and Xu 2011). In *C. elegans*, perlecan does not appear to be a generic BM component (Mullen *et al.* 1999) but is enriched at surfaces of contractile tissues, and is required for muscle-epidermal attachment (Francis and Waterston 1991; Hresko *et al.* 1994; Williams and Waterston 1994).

In addition to these core structural components, BMs have additional components that have more regulatory or tissue-specific roles. For example, in *C. elegans* the specialized BM of the somatic gonad is required for normal gonad development and migration, and contains gonad-specific components such as fibulin, nidogen, and ADAMTS proteases (Kubota *et al.* 2008, 2012). In contrast, the specialized BM that transduces force from the body wall muscles to the epidermis contains a distinct subset of proteins, including the F-spondin SPON-1 (Woo *et al.* 2008). Here we focus on the epidermal-muscle BM as it relates to embryonic elongation.

Embryonic epidermal morphogenesis and elongation in *C. elegans* involves multiple tissue types (Chisholm and Hardin 2005). Following gastrulation and the enclosure of internal tissues of the embryo by the epidermis in midembryogenesis, the embryo elongates into the tubular shape of larval and adult worms. The epidermis dramatically changes shape during elongation, extending almost threefold in the anterior–posterior axis (Priess and Hirsh 1986). Elongation requires epidermal cytoskeletal proteins (Ding *et al.* 2004), as well as muscle contraction and an intact epidermal-muscle BM (Williams and Waterston 1994; Chisholm and Hardin 2005).

We previously reported that the *C. elegans* extracellular matrix peroxidase PXN-2/peroxidasin is essential for embryonic development and elongation (Gotenstein *et al.* 2010). Peroxidasin was originally identified as a novel matrix peroxidase expressed in *Drosophila* development (Nelson *et al.* 1994). Biochemical studies revealed that peroxidasin can catalyze formation of the sulfilimine bonds required for type IV collagen network stabilization (Vanacore *et al.* 2009; Bhave *et al.* 2012). In *C. elegans*, PXN-2/peroxidasin-deficient animals arrest in late embryonic to early larval development, predominantly due to severe muscle-epidermal detachment and aberrant epidermal morphogenesis (Gotenstein *et al.* 2010). At the muscle-epidermal interface, *pxn-2* mutants develop an expanded extracellular matrix, containing multiple electron-dense basement-membrane-like layers. In *Drosophila* and zebrafish peroxidasin mutants, mechanical forces generated during tissue and organ development cause dissociation of BM and result in failed morphological development (Bhave *et al.* 2012, 2017; Fidler *et al.* 2014). Taken together, genetic analysis of peroxidasins is consistent with their proposed biochemical function in type IV collagen cross-linking.

In *C. elegans* body wall muscles, dense bodies and M lines are sites of integrin-based muscle adhesions (Francis and Waterston 1991), and are enriched for the BM components UNC-52/perlecan (Rogalski *et al.* 1993) and EPI-1/laminin αB (Huang *et al.* 2003). SPON-1 also localizes to sites of integrin adhesion; *spon-1* mutants arrest in late embryonic to early larval stages due to severe muscle detachment and epidermal elongation defects (Woo *et al.* 2008). SPON-1 does not appear to be required for laminin assembly; instead, SPON-1 may be required for proper integrin-mediated adhesion to the BM. The molecular mechanism of SPON-1 or F-spondin function is not yet understood.

In mammals, α6β4 integrin and collagen XVII/BP180 cooperatively link the epithelium to BM (Hirako and Owaribe 1998; Zhang and Labouesse 2010). However, the nature of the epidermal cell-matrix attachment in *C. elegans* has remained unclear. *C. elegans* encodes a single-pass transmembrane protein, myotactin/LET-805, which localizes to the basal side of the epidermis at structures known as fibrous organelles (Hresko *et al.* 1999). LET-805 has an extracellular domain (ECD) consisting of numerous fibronectin type III (FNIII) repeats, and is distantly related to FNIII-repeat proteins of the Sidekick family. Null mutations in *let-805* result in defective epidermal elongation and fully penetrant embryonic arrest (Hresko *et al.* 1999). Based on its molecular architecture, localization, and phenotypes, it has been hypothesized that LET-805 might be required for strong attachment of the epidermis to underlying BM. Moreover, both LET-805 and Perlecan display dosage sensitivity in certain genetic backgrounds (Zahreddine *et al.* 2010). It remains unclear if LET-805 interacts with specific ligands or whether it transduces signals into the epidermis.

Here, we report the results of large-scale screens for suppression of embryonic lethality in *pxn-2* or *spon-1* mutant backgrounds. We discovered multiple suppressor mutations clustered in a small region of the LET-805 ECD. We also identified missense suppressor mutations in the core BM components type IV collagen and perlecan. Genetic analysis is consistent with many of these acting as gain-of-function alleles. These genetic results suggest that compensatory mechanisms exist within the secreted and membrane-bound BM components that allow the bypass of otherwise essential functions.

## Materials and Methods

### Genetics and strain construction

*C. elegans* strains were maintained on NGM agar plates at 20–23° following standard procedures. Bristol N2 was used as wild type for all crosses and scoring. We used the following previously reported mutations and transgenes: *pxn-2*(*tm3464*), *pxn-2*(*ju358*, *ju432*, *ju436*), *pxn-1*(*ok785*), *spon-1*(*ju430*ts, *e2623*), *pxn-2+*(*juEx1044*), P*pxn-2*-PXN-2(*juEx2140*) (Gotenstein *et al.* 2010); *vab-10*(*e698*), *vab-19*(*e1036*cs) (Ding *et al.* 2003), *emb-9*::*Dendra* (*qyIs161*) (Ihara *et al.* 2011); *emb-9*(*b189*ts), *emb-9*(*g23cg46*) (Gupta *et al.* 1997), *let-2*(*b246*) (Sibley *et al.* 1994) *let-2*(*k193*), *let-2*(*k196*) (Kubota *et al.* 2008). Strains used are listed in Supplemental Material, Table S1 in File S1.

*vab-10*(*ju958*) arose as a spontaneous suppressor in a *pxn-2*(*ju358*) strain. *let-805* suppressors *ju1122* and *ju1123* were isolated in a semiclonal screen of ∼20,000 EMS mutagenized haploid genomes for suppression of the null *pxn-2*(*tm3464*) mutant to viability in the absence of the *pxn-2*(*+*) *juEx1044* GFP^+^ rescuing transgenic array. Suppressors *ju1165–ju1198* were isolated from a nonclonal screen for EMS-induced suppressors of lethality of the temperature sensitive allele *spon-1*(*ju430*ts). After mutagenesis, F_2_ and F_3_ animals were shifted from the permissive (15°) to the nonpermissive (25°) temperature. Surviving animals were propagated to generate suppressed mutant lines. Approximately 40,000 haploid genomes were screened.

### CRISPR mutagenesis and GFP knock-in

Single-guide RNA Cas9 vectors were cloned using the vector Addgene #47549 and the Quikchange mutagenesis protocol as described (Dickinson *et al.* 2013), except that Phusion hi-fidelity enzyme and buffer were substituted for Q5; see Table S3 in File S1 for targeting sequences. Point mutations were made using the *dpy-10*(*cn64*) co-CRISPR strategy as described (Arribere *et al.* 2014). A 121 bp oligonucleotide (Ultramer DNA; Integrated DNA Technologies) containing the desired change with 60 bp homology arms was used as repair template for the induced SNPs. Deletions *ju1299* and *ju1300* were generated in the course of attempting to recreate the *vab-10*(*ju958*) SNP. Recreation of the *ju1123* T/C (C1424R) SNP to generate *ju1386* used the *dpy-10*(*cn64*) co-CRISPR strategy with *let-805*(*ju1123*) single-guide RNA and oligo repair template.

GFP was inserted at the C-terminus of *let-805* in N2 (wild type) and suppressor alleles CZ19652 *ju1123*, CZ26231 *ju1165*, and CZ25774 *ju1184* using the Self-Excising Drug Selection Cassette as described (Dickinson *et al.* 2015). Homology arms were cloned into the pDD282 GFP-SEC-3xFlag vector to insert GFP directly before the stop codon. An *Avr*II/*Spe*I digest was used to include a flexible linker (RGASGASGAS) before the GFP sequence.

### LET-805::GFP image analysis

For each strain, adult worms grown at 20° for 24 hr after L4 stage we immobilized on 10% agarose pads. We took 5 µm, six-slice z-stack images of the midbody region centered on the vulval/uterine lumen on a Zeiss710 confocal microscope and exported as a maximum intensity projection. LET-805::GFP aggregation at the uterine seam was quantified using ImageJ particle analysis. A total of 12 images of each genotype were analyzed at 4 µm from the GFP localization at the uterine lumen. A region of interest of 30 × 120 pixels (∼13 × 52 µm) was used on each side of the uterine seam. A minimum threshold of 35 and a maximum of 255 were used to detect particles. Combined total area of all identified particles in each region of interest is reported.

### Transgenes

The following extrachromosomal transgenes were generated by injections of fosmid or plasmid DNA into N2 worms with P*ttx-3*-RFP as a co-injection marker: *juEx3911*–*juEx3915* contain *let-2* fosmid WRM0640bA06 (10 ng/μl), *juEx4356*–*juEx4360* contain *emb-9* fosmid WRM0610dH07 (10 ng/μl), and *juEx4361*–*juEx4365* were made by injecting a mix of the above *emb-9* and *let-2* fosmids (10 ng/μl each).

### Whole genome sequencing and genetic mapping

Linkage mapping of the *pxn-2* suppressor *ju958* was performed by crossing *pxn-2*(*tm3464*); *ju958* suppressed worms to the Hawaiian strain CB4856. *ju958* was mapped to chromosome I between N2/CB4856 SNPs located at ∼11,550 kb (+9.17 cM) and 12,950 kb (+15.72 cM). *vab-10* is at 11,750 kb (+9.62 cM). Worm genomic DNA was prepared for sequencing following the Gentra Puregene Tissue Kit DNA purification protocol. Whole genome sequencing (WGS) was performed by BGI, and analyzed as described (McCulloch *et al.* 2017). Strains were sequenced in batches, using the most outcrossed version of each suppressor available at the time. After the identification of the *let-805* “hotspot” regions, all suppressor strains were sequenced specifically in these regions and five new *let-805* alleles identified this way (Table 1). Strains in which no mutation was detected in the *let-805* hotspots were prepared for WGS.

**Table 1:** Summary of suppressors

Gene	Allele	Chromosome	Residue change	Codon change (wild type/mutant)	Mutation identification*^a^*/strain	% Lethality in *ju430**^b^*	% Lethality in *tm3464*
—	—	—	—	—	—	99.8	100
*vab-10*	*ju958*	I	D941N	Gat/Aat	WGS/mapping CZ13802	79.3	55.1
*vab-10*	*ju1299*	I	ΔD929-A937	27 bp Δ	CRISPR	ND	(31.2 in *ju358**^c^*)
*vab-10*	*ju1300*	I	ΔR931-D941	33 bp Δ	CRISPR	ND	(35.1 in *ju358**^c^*)
*unc-52*	*ju1168*	II	C402Y	tGc/tAc	WGS CZ21257	6.0	2.4
*unc-52*	*ju1172*	II	R1442C	Cgt/Tgt	WGS CZ23560	51.0	11.4
*unc-52*	*ju1174*	II	R175C	Cgc/Tgc	WGS CZ21494	36.5	19.0
*unc-52*	*ju1188*	II	E1480K	Gag/Aag	WGS CZ22297	ND	ND
*emb-9*	*ju1197*	III	P141L	cCa/cTa	WGS CZ22017	37.2	33.8
*let-2*	*ju1166*	X	E480K	Gaa/Aaa	WGS CZ20580	11.0	29.2
*let-2*	*ju1180*	X	P588L	cCa/cTa	WGS CZ21496	31.2	23.1
*let-805*	*ju1122*	III	E1547K	Gaa/Aaa	WGS CZ18264	38.5	20.6
*let-805*	*ju1123*	III	C1424R + R1584K	Tgc/Cgc + aGa/aAa	WGS CZ18265	25.1	6.9
*let-805*	*ju1386*	III	C1424R	Tgc/Cgc	CRISPR	24.3	1.7
*let-805*	*ju1165*	III	S1594F	tCc/tTc	WGS CZ20579	8.9	4.8
*let-805*	*ju1167*	III	G1541E	gGa/gAa	WGS CZ20581	33.7	ND
*let-805*	*ju1170*	III	R1076C	Cgt/Tgt	WGS CZ20988	36.8	36.2
*let-805*	*ju1173*	III	R970C	Cgt/Tgt	WGS CZ20990	9.3	61.1
*let-805*	*ju1175*	III	R1443C	Cgt/Tgt	Resequencing	44.8	ND
*let-805*	*ju1177*	III	G1541R	Gga/Aga	Resequencing	32.1	ND
*let-805*	*ju1184*	III	G1578E	gGa/gAa	Resequencing	4.5	32.6
*let-805*	*ju1187*	III	P1422R	cCa/cGa	WGS CZ22296	29.0	2.5
*let-805*	*ju1190*	III	V1052I	Gtc/Atc	Resequencing	47.1	ND
*let-805*	*ju1198*	III	A1552V	gCt/gTt	Resequencing	37.4	ND

a WGS, Whole genome sequencing. Resequencing, Sanger sequencing of *let-805* hotspots.

b at 25°.

c *ju358* is a viable, strong loss-of-function allele of *pxn-2*. The single mutant displays 58.5% lethality.

### Phenotypic analysis of suppression

We quantitated the penetrance of lethal and epidermal morphology defects essentially as described (George *et al.* 1998). For each genotype, the entire self-progeny brood of P_0_ animals was quantified (*n* = 5). *P* values were determined for total lethality using Student’s *t*-test, assuming two-tailed unequal variance. To test heterozygous suppression of *pxn-2*(*tm3464*), we crossed homozygous suppressed males to transgenically rescued null mutants, *pxn-2*(*tm3464*); *juEx1044*. Broods of five nontransgenic F_1_ progeny were quantified at 20° unless specified. In the case of *let-2*(*ju1166/+*), we were unable to isolate viable F_1_ heterozygotes, so broods of nontransgenic F_2_s from *let-2*(*ju1166/+*); *pxn-2*(*tm3464*);*juEx1044* parent worms were quantified and genotyped to confirm the heterozygosity of parent worms. For complementation testing, single hermaphrodites were mated to homozygous males; four single hermaphrodite crosses were quantified. Quantitative data are in Table S1 in File S1. Suppression of pharynx BM defects was assessed by DIC analysis of day 1 adult worms grown at 20°. Worms were analyzed for bulging in either bulb of the pharynx.

### RNA interference

We performed RNA interference (RNAi) by feeding. Worms were grown at 20° throughout. L4 worms transferred to 5 mM IPTG, 50 µg/ml carbenicillin plates seeded with RNAi bacteria. After 24 hr, three adult worms per genotype and per clone were transferred to individual plates. After 24 hr, parent worms were removed from plates. Progeny were counted and phenotypes assessed after an additional 24 hr. We used clones from the Ahringer library: clone III-1J01 for *let-805* and clone III-5A13 for *emb-9* (Kamath *et al.* 2003). A GFP RNAi clone (gift from E. Troemel, University of California, San Diego) was used to target LET-805::GFP. For the lethal *pxn-2*(*tm3464*), parent worms expressing a transgenic extrachromosomal array (*juEx1044*) of cosmid K09C8 (*pxn-2*) + P*sur-5*-GFP were plated. Only nontransgenic progeny were assessed.

### Protein sequence alignments and structural modeling

We performed alignments of LET-805 with other FNIII repeat-containing proteins using BLAST. The numbering of FNIII repeats in Figure S2 in File S1 is based on reanalysis with additional protein domain prediction algorithms; sequence alignments use ClustalX. Protein domain graphics were generated using IBS software (Liu *et al.* 2015), based on domains identified in the conserved domain database of the National Center for Biotechnology Information (Marchler-Bauer *et al.* 2015). Predicted structures of subsets of the LET-805 FNIII repeats were generated using SWISS-MODEL (Arnold *et al.* 2006). Models in Figure S3 in File S1 are based on the human IL-6 receptor FNIII repeats.

### Data availability

The authors state that all data necessary for confirming the conclusions presented in the article are represented fully within the article.

## Results

### Identification of a novel mutation in spectraplakin *vab-10* that suppresses developmental defects of peroxidasin *pxn-2* mutants

**Figure 1:**
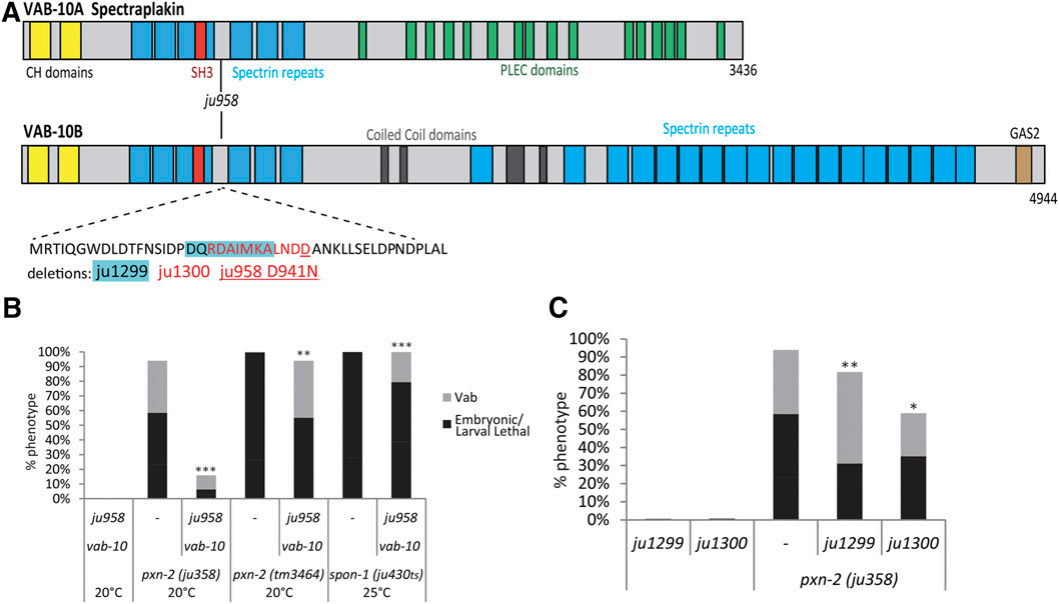
Suppression of BM mutants by a spontaneous mutation in *vab-10*. (A) Structure of VAB-10 A and B protein domains with *ju958* D941N and deletions noted. (B) *vab-10*(*ju958*) suppresses *pxn-2* null to viability; *ju958* partially suppresses *spon-1*(*ju430ts*) lethality at nonpermissive temperature. (C) Small in-frame deletions generated by CRISPR near the *vab-10*(*ju958*) missense residue partly phenocopy the suppression of *pxn-2*(*ju358*) by *ju958*. Results of Student’s *t*-test for percent total lethality (sum of embryonic and larval lethality). * *P* < 0.05, ** *P* < 0.01, *** *P* < 0.001.

We previously reported that *C. elegans pxn-2* mutants have defective BMs, leading to aberrant tissue morphogenesis and lethality (Gotenstein *et al.* 2010). *pxn-2*(*ju358*) is a partial loss of function allele that causes ∼60% embryonic and larval lethality and severe morphology defects (Gotenstein *et al.* 2010); *pxn-2*(*ju358*) homozygotes can be propagated as slow-growing strains. We identified a fast-growing variant strain in the course of routine propagation and confirmed that it retained the original *pxn-2*(*ju358*) mutation. This suppressed strain, *pxn-2*(*ju358*) *sup*(*ju958*), displays <5% lethality and almost no detectable morphological defects. Following outcrossing and genetic mapping, we showed that the suppressor *ju958* mapped to a 1.4 Mb region on chromosome I. WGS analysis revealed a single missense alteration in this region, affecting *vab-10*. *vab-10* encodes two sets of spectraplakin isoforms, VAB-10A and VAB-10B, each essential for embryonic morphogenesis and epidermal integrity (Bosher *et al.* 2003). The *ju958* missense mutation alters residue D941N in a linker between N-terminal spectrin repeats common to VAB-10A and VAB-10B (Figure 1A). In contrast to *vab-10* loss-of-function alleles, *ju958* single mutants were fully viable and displayed normal morphology.

We found that *ju958* was able to suppress the *pxn-2*(*tm3464*) null allele [hereafter, *pxn-2*(*0*)] to viability (Figure 1B). We previously reported suppression of *pxn-2* by the superficially wild-type *pxn-1*(*ok785*) null mutation (Gotenstein *et al.* 2010). Triple mutant *vab-10*(*ju958*); *pxn-1*(*ok785*); *pxn-2*(*0*) animals showed additive suppression of *pxn-2* (Table S1 in File S1), suggesting that the mechanisms of suppression by *pxn-1* and by *vab-10*(*ju958*) are distinct. To test specificity of the suppression for BM defects we tested an additional BM mutant, *spon-1*(*ju430*ts), and found that *ju958* partially rescues the conditional lethality of *spon-1* (Figure 1B). Thus, suppression by *vab-10*(*ju958*) is not gene specific.

The *vab-10* locus extends over 45 kb, precluding standard transgenic rescue or reconstruction experiments. To confirm whether the *vab-10*(*D941N*) missense alteration was causative for suppression, we performed CRISPR-mediated mutagenesis of wild-type animals at the *vab-10*(*D941*) locus and isolated two small, in-frame deletions adjacent to or encompassing the residue affected in *ju958* (Figure 1A). These deletions, *ju1299* and *ju1300*, partially suppressed phenotypes of *pxn-2*(*ju358*) (Figure 1C), although they did not suppress the *pxn-2* null allele to viability. As these deletions also showed suppression of *pxn-2*(*ju358*), we conclude that *vab-10* mutations in this region are causative for suppression of *pxn-2* loss of function.

### Suppressor screening of *pxn-2* and *spon-1* identified novel mutations in components of the BM and cell-matrix attachments

Encouraged by the finding that *pxn-2* null mutant phenotypes can be suppressed to viability, we screened directly for EMS-induced suppressors of *pxn-2*(*0*) phenotypes (see *Materials and Methods*) and recovered two suppressors that were identified as alleles of *let-805*. As described below, these *let-805* alleles also suppressed lethal phenotypes of *spon-1*/F-spondin. We therefore took advantage of the temperature sensitive lethality of a partial loss-of-function allele, *spon-1*(*ju430*ts), to perform a larger-scale selection for suppressors (see *Materials and Methods*). We recovered 34 additional suppressors of *spon-1*(*ju430*ts). By mapping and WGS, we identified 10 additional alleles of *let-805*/myotactin, three affecting *emb-9* or *let-2* (type IV collagen) and four affecting *unc-52*/perlecan (Table 1). Seventeen additional suppressors will be described elsewhere.

### Peroxidasin *pxn-2* displays specific genetic interactions with type IV collagen mutations and transgenes

**Figure 2:**
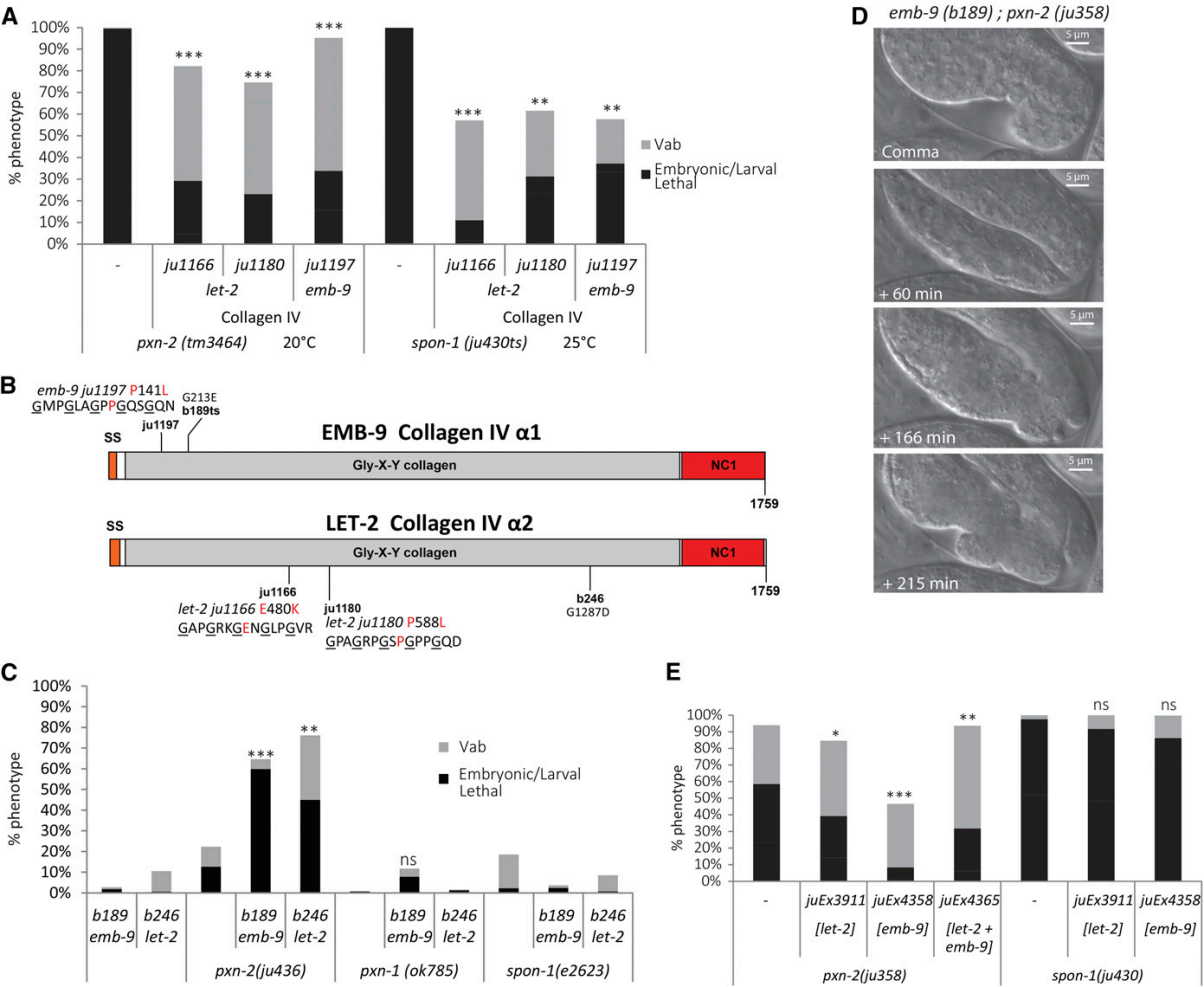
Genetic interactions of type IV collagen mutants with peroxidasin *pxn-2*. (A) Novel type IV collagen alleles suppress both *pxn-2*(*tm3464*) null and *spon-1*(*ju430ts*) to viability. (B) Structure of EMB-9 and LET-2 proteins with location of alleles annotated. Novel type IV collagen alleles: *ju1166* causes E480K in LET-2, *ju1180* causes P588L in LET-2, and *ju1197* causes P141L in EMB-9. (C) Loss of function in *pxn-2* synergistically enhances the lethality of weak type IV collagen *let-2* or *emb-9* mutants. A null mutation in *pxn-1* does not show synergistic enhancement with type IV collagen alleles. Additionally *spon-1* loss of function is unaltered by type IV collagen loss of function. (D) Time series of *emb-9*(*b189*); *pxn-2*(*ju358*) embryo from comma to arrest. (E) Transgenes containing type IV collagen DNA can partially rescue lethality of *pxn-2*(*lf*), but not *spon-1*(*lf*). Results of Student’s *t*-test are indicated for percent total lethality (sum of embryonic and larval lethality). * *P* < 0.05, ** *P* < 0.01, *** *P* < 0.001.

*C. elegans* type IV collagen is composed of heterotrimers of two α1-like (EMB-9) and one α2-like (LET-2) chains (Kramer 2005). Of the three suppressor mutations we identified as affecting type IV collagen chains, two cause missense alterations in LET-2 and one affects EMB-9. In contrast to previously reported loss- or gain-of-function alleles of *C. elegans* type IV collagen (Sibley *et al.* 1994; Kubota *et al.* 2008), these suppressor alleles do not cause detectable phenotypes as single mutants, and suppress the lethality of *pxn-2* null and of *spon-1* loss of function (Figure 2A). All three affect non-Gly residues within the Gly-X-Y repeats of the collagenous α-helical domain; two cause Pro-to-Leu missense changes (Figure 2B). In humans, mutations in type IV collagen are known to cause dominantly inherited cerebrovascular disease; a Pro-to-Leu missense change in *COL4A1* has been identified in a patient with sporadic cerebral hemorrhage (Weng *et al.* 2012). This study observed increased intracellular aggregation of this type IV collagen variant, similar to the effects of mutations affecting Gly residues in the Gly-X-Y repeats. In *C. elegans*, complete loss of function in *let-2* or *emb-9* causes fully penetrant embryonic lethality, so suppressor alleles of these genes might be either very weak loss of function or gain of function. *emb-9*(*ju1197*) fully complemented the lethality of *emb-9* null mutants (Table S1 in File S1), consistent with a gain of function. Moreover, *emb-9* RNAi abrogated the suppression of *pxn-2*(*0*) by *emb-9*(*ju1197*) at RNAi dosages that do not cause defects in a wild-type background or enhancement of *pxn-2*(*0*) (Figure S1A in File S1). *let-2*(*ju1166*)*/^+^* heterozygotes displayed weak suppression (Figure S1B in File S1). Overall, our analysis is most consistent with a gain-of-function model for these suppressor alleles of type IV collagen.

Given the findings that insect and mammalian peroxidasins can promote formation of sulfilimine bonds between type IV collagen NC1 domains *in vitro* (Bhave *et al.* 2012), we sought genetic evidence that *C. elegans pxn-2* functions in type IV collagen cross-linking *in vivo*. If PXN-2 functions to promote type IV collagen cross-linking, loss of function in *pxn-2* and type IV collagen should synergize genetically. The temperature-sensitive allele *emb-9*(*b189*) causes 100% lethality at the restrictive temperature 25°, although it is 98% viable at 20°. When combined with *emb-9*(*b189*), the relatively weak allele *pxn-2*(*ju436*) caused a 30-fold increase in lethality at 20° (Figure 2C). We were unable to test interactions at 25°, as surviving double mutants were sterile. The strongest viable allele *pxn-2*(*ju358*) was 100% lethal in combination with *b189*: double mutants arrested at the threefold stage of embryogenesis (Figure 2D), similar to *pxn-2* (Gotenstein *et al.* 2010) and *emb-9* (Gupta *et al.* 1997) null mutants. Type IV collagen mutant phenotypes were not modified by loss of function in the other *C. elegans* peroxidasin gene *pxn-1* (Figure 2C).

We observed a similar synergism between a weak allele of α2 type IV collagen, *let-2*(*b246*), with the hypomorphic allele *pxn-2*(*ju436*). Double mutant strains were nearly 50% lethal, whereas single mutants displayed minimal defects. In addition, we tested interaction of a reported type IV collagen gain-of-function allele, *let-2*(*k193*), which alters an amino acid residue in the NC1 domain, with an intermediate loss-of-function allele *pxn-2*(*ju432*) to potentially detect either suppression or synergy. *let-2*(*k193*) affects gonad development but does not cause embryonic lethality (Kubota *et al.* 2008). This type IV collagen allele strongly enhanced *pxn-2*(*ju432*) lethality (Table S1 in File S1), similar to the type IV collagen loss-of-function alleles. In contrast, type IV collagen loss-of-function mutations did not display synergistic lethality with a weak allele of *spon-1e2623* (Figure 2C). Collectively, these genetic interactions support the specific synergy of type IV collagen with PXN-2.

We also tested whether overexpression of type IV collagen might bypass the need for *pxn-2*. Among multiple transgenes tested, we found that a transgenic array of *emb-9*(*+*) fosmid significantly reduced the lethality and morphological defects of a partial loss-of-function mutant *pxn-2*(*ju358*) (Figure 2E), although this array was not able to suppress *pxn-2* null mutant lethal phenotypes. Transgenes containing the *let-2*(*+*) fosmid alone or a combination of *let-2* and *emb-9* fosmids were less effective at suppressing *pxn-2*(*ju358*) phenotypes (Figure 2E), likely due to the 2 EMB-9:1 LET-2 composition of type IV collagen. In contrast, transgenic arrays expressing type IV collagen were unable to suppress *spon-1*(*ju430*ts) phenotypes (Figure 2E), suggesting SPON-1 does not directly affect type IV collagen function. Together with our double mutant analyses, these genetic interactions are consistent with PXN-2 playing a specific role in type IV collagen function *in vivo*.

### Gain-of-function mutations in a critical region of the LET-805 ECD suppress *pxn-2* and *spon-1* null mutant phenotypes

**Figure 3:**
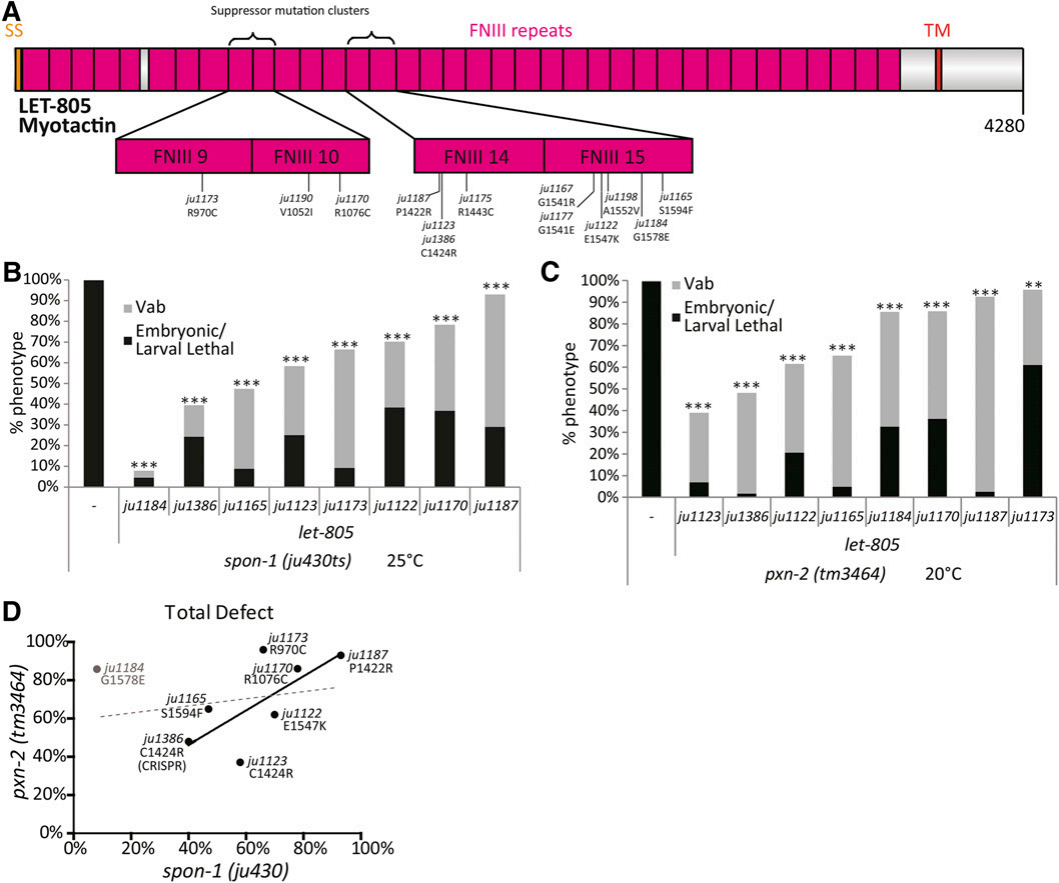
Missense mutations in the *let-805* ECD suppress phenotypes due to loss of function in multiple components of the BM. (A) Domain structure of LET-805, indicating the two pairs of FNIII repeats affected in suppressor alleles. See also Figure S2 in File S1. (B) Level of suppression of *spon-1*(*ju430*) lethality and Vab phenotypes by *let-805* alleles at 25°. (C) Level of suppression of *pxn-2*(*tm3464*) null mutant lethality and Vab phenotypes by *let-805* alleles at 20°. (D) Correlation of strength of suppression of *spon-1* *vs.* *pxn-2* by *let-805* suppressors. Solid line shows correlation trend line excluding *ju1184* (slope = 0.86 ± 0.40); dotted line shows correlation trend line including *ju1184* (slope = 0.19 ± 0.33). Results of Student's *t*-test for percent total lethality (sum of embryonic and larval lethality). ** *P* < 0.01, *** *P* < 0.001.

We identified 12 suppressor mutations as causing missense alterations in LET-805/myotactin (Figure 3A). The *let-805* gene encodes a predicted single-pass transmembrane protein containing fibronectin type III (FNIII) repeats in its ECD (Hresko *et al.* 1999). Through sequence alignment and domain analysis (see *Materials and Methods*), we identified four additional FNIII repeats as well as the 32 previously annotated (see Figure S2 in File S1). Strikingly, all 12 of the identified *let-805* suppressor alleles affect two pairs of adjacent FNIII repeats (repeats 9+10 and 14+15, as numbered in Figure 3A and Figure S2A in File S1) in the middle of the ECD. Although the three alleles located in FNIII repeats 9+10 were generally weaker suppressors than mutations affecting repeats 14+15, overall these *let-805* mutations displayed strong suppression of lethality of null or loss-of-function alleles of both *spon-1* and *pxn-2* (Figure 3, B and C and Figure 5). Moreover, *ju1123* displayed semidominant suppression of *pxn-2*(*0*) (Figure S1C in File S1). As loss of function in *let-805* causes fully penetrant recessive embryonic lethality (Hresko *et al.* 1999), we hypothesize *ju1123* and other suppressor alleles of *let-805* cause a gain of function. Consistent with this interpretation, *let-805* RNAi by feeding, which does not cause lethality in a wild-type background, abrogated suppression in a *let-805*(*ju1123*); *pxn-2*(*0*) background (Figure S1, D and E in File S1).

To confirm that the *let-805* missense changes were causative for genetic suppression, we used CRISPR to recreate the base transition causing the C1424R alteration, one of two missense changes in the original *let-805*(*ju1123*) suppressor strain. The resulting mutant, denoted *ju1386*, suppressed both *spon-1* and *pxn-2* phenotypes to levels equivalent to the original *let-805*(*ju1123*) suppressor (Figure 3, B and C). These observations confirm that missense alterations in the LET-805 ECD can suppress the requirement for *pxn-2* or *spon-1* in development.

PXN-2 and SPON-1 likely have distinct molecular roles in the BM; consistent with this, *pxn-2* but not *spon-1* was suppressed by transgenic overexpression of type IV collagen (Figure 1F). Nevertheless it is striking that almost all the *let-805* alleles isolated as suppressors of *spon-1*(*ju430*ts) were subsequently found to suppress *pxn-2* null phenotypes, suggesting a correlation between suppression of *pxn-2* and *spon-1*. We compared the relative level of suppression of *spon-1* and *pxn-2*, using eight *let-805* alleles. Overall there is a correlation between suppression of *spon-1* with suppression of *pxn*-2 (Figure 3D), with the exception of *let-805*(*ju1184*), which strongly suppresses *spon-1* phenotypes but weakly suppresses *pxn-2*. If *ju1184* is excluded, suppression of the two loci is strongly positively correlated, suggestive of a common mechanism of suppression.

To understand how these missense alterations might affect LET-805 structure, we modeled the four FNIII repeats affected by the suppressor mutations (see *Materials and Methods*) (Figure S3 in File S1). The predicted structures are consistent with these repeats forming a binding interface that is affected by the suppressor missense mutations. The missense alterations could directly alter the affinity of LET-805 for an as-yet unidentified ligand. Alternatively, mutations in this region of the ECD could result in a conformational change that alters LET-805 signaling independent of ligands.

**Figure 4:**
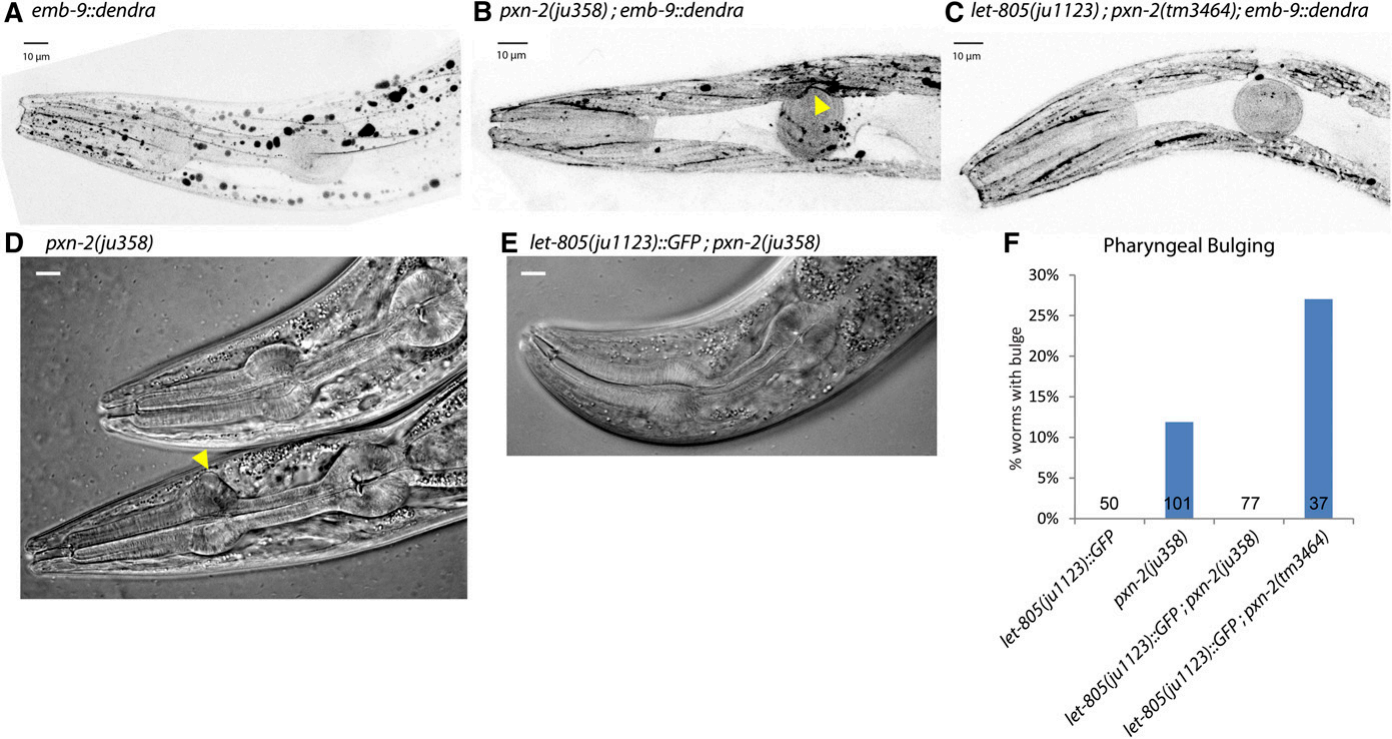
Suppression of BM defects by *let-805*(*ju1123*). Functional EMB-9::Dendra (*qyIs161*, fluorescence is shown as inverted, grayscale, projections of z-stacks of superficial sections) localizes to BM overlying body wall muscles and pharynx, as well as accumulating in intracellular aggregates, in wild type (A) and in *let-805*(*ju1123*) (data not shown). In *pxn-2*(*ju358*), the normal linear localization of EMB-9::Dendra is interrupted (B, arrowhead). (C) EMB-9::Dendra localization is essentially normal in suppressed *let-805*(*ju1123*); *pxn-2*(*tm3464*) animals. (D–F) Distortions in pharyngeal morphology of *pxn-2* young adults (arrowhead, D) are completely suppressed by *let-805*(*ju1123*) (image, E and quantitation, F). Scale bars, 10 microns.

Suppression of *pxn-2* or *spon-1* lethal phenotypes could be for a number of reasons. We therefore examined whether specific *pxn-2* phenotypes attributed to BM defects were altered by the *let-805* suppressors. Four-dimensional time-lapse analysis of *let-805*(*ju1123*) *pxn-2*(*0*) animals confirmed that suppressed animals showed restored epidermal morphology and body wall muscle attachment (data not shown). In adults, we observed type IV collagen and found distortions in the structure of BM tracks in *pxn-2*(*ju358*) compared to wild type (Figure 4, A and B). These distortions were eliminated in suppressed *let-805*(*ju1123*) *pxn-2*(*0*) animals (Figure 4C). Furthermore, the progressive distortions in pharyngeal morphology of *pxn-2*(*ju358*) hypomorphs were fully suppressed by *let-805*(*ju1123*) (Figure 4, D–F). Taken together, these analyses are consistent with *let-805* alleles suppressing multiple *pxn-2* mutant phenotypes that result from aberrant BM function.

### *let-805* alleles suppress phenotypes due to partial loss of function in the BM, but not due to loss of function in the epidermal cytoskeleton

**Figure 5:**
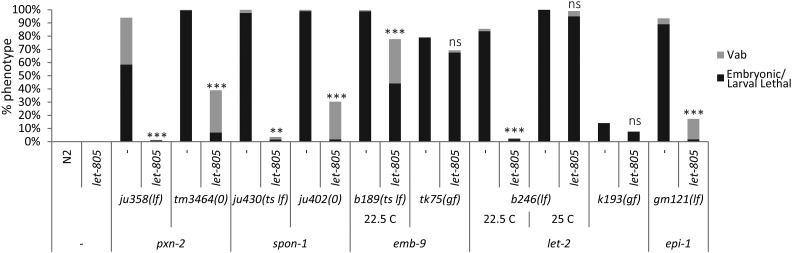
*let-805*(*ju1123*) suppresses lethality and morphological defects due to partial loss of function in core structural BM components, including the type IV collagen chains *emb-9* and *let-2* and the laminin subunit *epi-1*. *let-805*(*ju1123*) did not suppress defects of *emb-9*(*tk75*) or *let-2*(*b246*) at the restrictive temperature. Results of Student’s *t*-test are indicated for percent total lethality (sum of embryonic and larval lethality). ** *P* < 0.01, *** *P* < 0.001.

Given the observed synergism of loss-of-function mutations in *pxn-2* with type IV collagen, we next tested whether *let-805* suppressor alleles could also suppress type IV collagen mutant phenotypes. We found that *let-805*(*ju1123*) partially suppressed the lethality of *emb-9*(*b189*ts) at the intermediate temperature of 22.5° (Figure 5), but did not suppress the sterility of *b189* mutants shifted to 25°. *let-805*(*ju1123*) also suppressed the lethality of *let-2*(*b246*ts) at the intermediate temperature of 22.5°, but did not significantly suppress *b246* at the restrictive temperature of 25° (Figure 5). *let-805*(*ju1123*) did not suppress either *emb-9*(*tk75gf*) or *let-2*(*k193gf*) (Figure 5). To address whether *let-805* suppressor alleles bypass the requirement for type IV collagen, we attempted to test a null allele *emb-9*(*g23cg46*) but were unable to generate a viable *let-805*(*ju1123*); *emb-9*(*g23cg46*) strain. We further found that *let-805*(*ju1123*) was able to suppress the phenotypes of a partial loss-of-function mutation in the laminin αB chain gene *epi-1*(*gm121*) (Forrester and Garriga 1997) (Figure 5). Taken together, these data indicate that the *let-805* gain-of-function mutations can suppress defects in BM integrity due to complete loss of function in nonstructural genes (*pxn-2*, *spon-1*) or partial loss of function in structural genes (type IV collagens, laminins), but are unable to bypass the requirement for structural BM components.

Given the suppression of BM mutant defects by *vab-10*(*ju958*), we also tested whether *let-805*(*ju1123*) suppressed mutant defects of epidermal cytoskeletal proteins including *vab-10* and other components of *trans*-epidermal attachments, where LET-805 is known to localize. Partial loss of function in genes encoding cytoskeleton components (including *vab-10*/spectraplakin, intermediate filament *ifb-1*, and *vab-19*/Kank) were not suppressed by *let-805* suppressor alleles (Figure S4 in File S1), suggesting suppression is specific to defects in BM components.

### The LET-805 gain-of-function mutations do not overtly affect protein expression level or localization, but cause aggregation of LET-805::GFP

**Figure 6:**
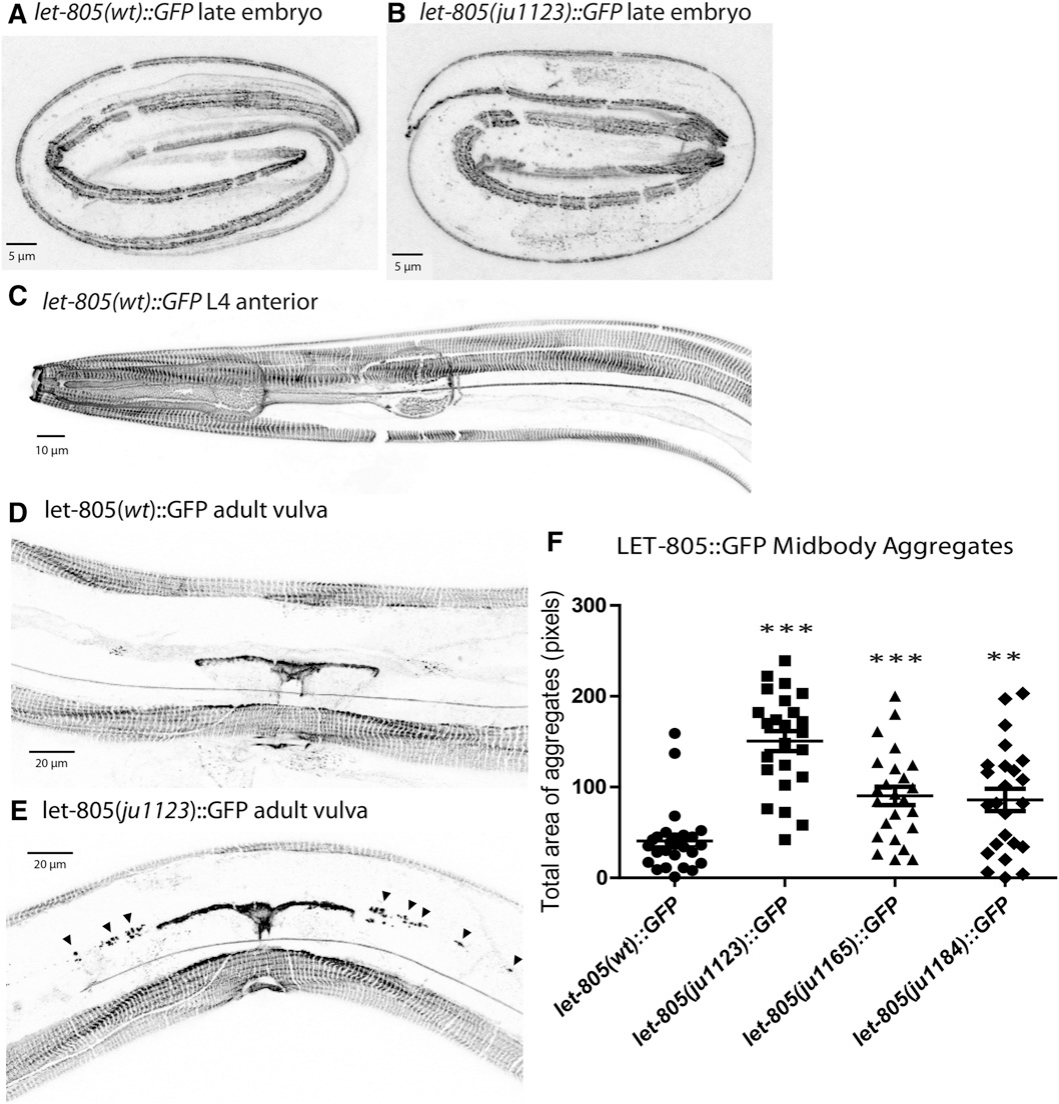
Localization of LET-805::GFP knock-ins. In late-stage embryos from (A) wild-type, (B) *let-805*(*ju1123*), and (C) larval animals, LET-805::GFP localizes to epidermal attachment structures overlying body wall muscles and (D) to the uterine seam. In embryos, LET-805::GFP localization in *let-805* suppressor mutant backgrounds resembled that of the wild-type LET-805::GFP; in adults mutant for suppressor alleles *let-805*(*ju1123*), *let-805*(*ju1165*), and *let-805*(*ju1184*), LET-805::GFP displays increased aggregates along the mid-body (E) (arrowheads). (F) Quantitation of aggregation as described in *Materials and Methods*; significance, *t*-test, ** *P* < 0.01, *** *P* < 0.001.

To assess whether *let-805* missense mutations affect LET-805 localization or levels, we used CRISPR to insert a C-terminal GFP tag in the wild-type, *ju1123*, *ju1165*, and *ju1184* mutant *let-805* loci (see *Materials and Methods*). All these GFP knock-in animals were fully viable. Wild-type LET-805::GFP localized to epidermal hemidesmosomes, from midembryogenesis onwards (Figure 6), as expected from prior immunocytochemical studies (Francis and Waterston 1991). We also observed localization at the pharynx and uterine seam, consistent with previous results (Francis and Waterston 1991), and along touch neuron processes (Figure 6). These are all regions of the epidermis that require strong adhesive coupling to internal cells. The expression and localization of LET-805(*ju1123*)::GFP in embryos was overall similar to that of wild-type LET-805::GFP. However, in adults the aggregation of LET-805(*ju1123*)::GFP at the uterine-epidermal contact was significantly increased compared to the wild type (Figure 6, D–F). Increased aggregation compared to wild type was seen in all three knock-in alleles (Figure 6F). We conclude that the suppressor missense changes do not dramatically affect LET-805 levels or localization, but might enhance its tendency to cluster, consistent with models whereby LET-805 undergoes dimerization or multimerization.

Wild-type LET-805::GFP was severely mislocalized in *spon-1* or *pxn-2* mutants (Figure S5 in File S1), consistent with the epidermal-muscle detachment observed in these animals (Woo *et al.* 2008; Gotenstein *et al.* 2010). The suppressor LET-805(*ju1123*)::GFP strongly restored localization and proper hemidesmosome morphology in *pxn-2* or *spon-1* mutants, from late embryos through to adults (Figure S5 in File S1), indicating that the GFP tag does not affect the gain of LET-805 function.

### Mutations in UNC-52 perlecan can suppress lethality of *pxn-2* and *spon-1* mutants

**Figure 7:**
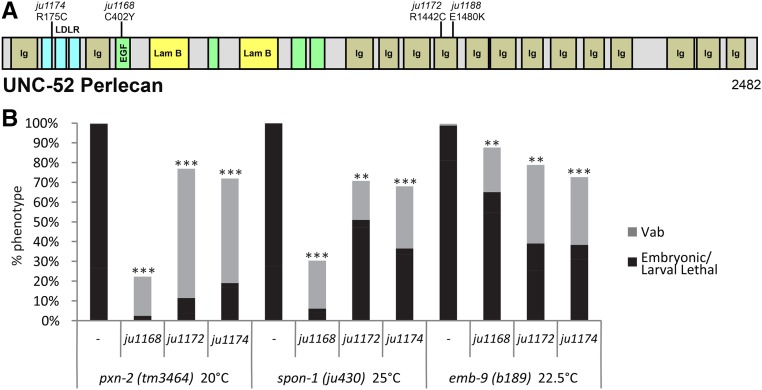
Suppression of BM phenotypes by missense alterations in *unc-52* perlecan. (A) Domain structure of UNC-52 (A isoform) and locations of mutations and amino acid substitutions. (B) *unc-52* suppressor alleles suppress *pxn-2*(*tm3464*) and *spon-1*(*ju430*) lethality, and weakly suppress *emb-9*(*b189ts*) lethality. LDLR, low-density lipoprotein receptor class A repeat; EGF, Laminin EGF-like domain; Lam B, Laminin B domain. Results of Student's *t*-test for percent total lethality (sum of embryonic and larval lethality). ** *P* < 0.01, *** *P* < 0.001.

We identified four additional suppressors of *spon-1* as affecting the *unc-52* gene, encoding the BM component perlecan (Figure 7A). Unlike *let-805* suppressor alleles, the *unc-52* suppressor alleles affect distinct functional domains across the protein; all four strongly suppress both *pxn-2* and *spon-1* mutant lethality (Figure 7B). The strongest suppressor *unc-52*(*ju1168*) affects a laminin-type EGF-like domain. The residue affected in *ju1168* is conserved with human HSPG2. In contrast, *unc-52*(*ju1174*) affects a nonconserved residue between a conserved Cys and the conserved DXSDE motif in the LDL receptor-like domain of perlecan. Loss of function in specific isoforms of UNC-52 results in adult locomotor defects (Unc) due to progressive loss of muscle structure (Rogalski *et al.* 1995). The final two suppressors affect an Ig domain of Perlecan domain IV. Many of the previously identified Unc mutants localize to this region (Mullen *et al.* 1999). However, unlike previously identified *unc-52* variants, the suppressor alleles do not display Unc or Let phenotypes, suggesting *unc-52* suppressor alleles may cause gain of function or weak loss of function. Consistent with a gain-of-function interpretation, *unc-52*(*ju1168*)*/^+^* heterozygotes displayed semidominant suppression of *pxn-2*(*0*) (Figure S1F in File S1). Conversely, the hypomorphic allele *unc-52*(*e444*) displayed additive phenotypes when combined with the weak allele *pxn-2*(*ju436*) (data not shown), suggesting loss of *unc-52* function does not modify *pxn-2* phenotypes.

The *unc-52* suppressor mutants also partially suppress loss of function in type IV collagen *emb-9*(*b189*) (Figure 7B), comparable to the *let-805* suppression of type IV collagen mutants (Figure 5). Previous studies have suggested myotactin/LET-805 and perlecan/UNC-52 function are closely related (Zahreddine *et al.* 2010). We therefore tested whether *let-805* suppressor alleles could suppress *unc-52* loss of function. The Unc phenotypes of the partial loss-of-function allele *unc-52*(*e444*) were unaltered by the *let-805*(*ju1123*) suppressor (Table S1 in File S1). We also tested the putative null allele, *unc-52*(*st549*) (Rogalski *et al.* 1995), which causes fully penetrant embryonic lethality, and found that this lethality was not suppressed by *let-805*(*ju1123*). This finding suggests that the requirement for certain BM components, such as perlecan, type IV collagen, and laminin, is not bypassed by the kinds of LET-805 altered function mutations described here.

## Discussion

Starting from the chance observation of extragenic suppression of *C. elegans* peroxidasin/*pxn-2* phenotypes, we have found that a variety of genes can be mutated to suppress otherwise lethal phenotypes of matrix mutants. Our genetic analysis of *pxn-2* supports the model that peroxidasins are critical for type IV collagen cross-linking. Nevertheless, the essential role of *pxn-2* can be bypassed by putative gain-of-function alterations in other essential BM components or in the putative matrix receptor LET-805. These suppressor alleles also suppress phenotypes due to loss of function in SPON-1. As PXN-2 and SPON-1 likely play different roles in the BM, such suppression may act at the overall level of matrix function rather than compensation for loss of a specific pathway or interaction. These genetic suppression interactions reveal a previously unknown capacity for compensatory interactions between these individually essential genes.

### Genetic interactions are consistent with peroxidasin and type IV collagen functioning in a common process

Our observations of synergism between loss-of-function mutations in the type IV collagen α1 *emb-9* and *pxn-2*, as well as rescue of *pxn-2* phenotypes by transgenic overexpression of type IV collagen, is consistent with PXN-2 promoting type IV collagen function in *C. elegans*. From other biochemical studies, peroxidasins are believed to catalyze sulfilimine cross-links between type IV collagen NC1 domains. In a *pxn-2* mutant background, formation of these sulfilimine bonds is presumably reduced, although our efforts to directly assess type IV collagen cross-linking have been unsuccessful, as the embryonic lethality of *pxn-2* mutants precludes biochemical analysis. We infer that the combination of partial reduction in type IV collagen cross-linking and partial loss of function in a type IV collagen chain results in severely compromised structural integrity of the BM and subsequent embryonic lethality.

The mechanism by which putative gain-of-function mutations in type IV collagen results in suppression of *pxn-2* remains uncertain. The strong suppression of *spon-1* argues against a direct bypass of the *pxn-2*–mediated sulfilimine bond. Given the location of the type IV collagen suppressor alleles in the collagenous domain, we speculate that the mutations may enhance lateral associations of parallel collagen chains (Yurchenco and Ruben 1987; Knupp and Squire 2005), potentially relaxing the requirement for peroxidasin-mediated cross-linking of the NC1 domains. Hydroxylated proline and/or lysine have been shown to increase the thermostability of collagen (Berg and Prockop 1973). By analogy, a general increase in type IV collagen stability could overcome the need for sulfilimine cross-linking and thus allow genetic suppression of *pxn-2* phenotypes.

### Role of myotactin/LET-805 in BM interactions

LET-805/myotactin is a giant transmembrane protein whose ECD is composed solely of FNIII repeats. Although myotactin is not conserved outside of nematodes, it has been proposed to play an analogous role to the hemidesmosome transmembrane protein BPAG2 (formerly BP180) (Hresko *et al.* 1999), which interacts directly with α6β4 integrin and with the intracellular plakin domains of plectin (Koster *et al.* 2003). As BPAG2 and myotactin show no sequence similarity, it is unclear whether they share any common mechanisms. It is intriguing that missense alterations in *let-805* and the spectraplakin VAB-10 can result in similar genetic suppression of BM defects

The suppressors identified as LET-805 missense changes alter a variety of amino acid residues of its FNIII repeats, in general resulting in either removal of or addition of a charged residue. All residues affected are conserved in the equivalent repeats of myotactin orthologs in other nematodes (Figure S2C in File S1), but are not conserved in the other FNIII repeats of *C. elegans* myotactin or of other Sidekick family members (Figure S2, A and B in File S1), suggesting these four FNIII repeats may have distinctive roles in myotactin function. The most distinctive features of these repeats are sequences of nonconserved residues between FNIII repeats 8 and 9, and in the middle of FNIII repeat 15 (underlined in red, Figure S2A in File S1). Linker regions between FNIII repeats are important for function in other molecules such as fibronectin itself (Vakonakis *et al.* 2007). As FNIII repeats are often in large clusters, there are scant examples of single missense alterations having phenotypic consequences. A missense alteration in a FNIII repeat in the PTPRQ transmembrane phosphatase has been linked to deafness (Schraders *et al.* 2010); how this mutation affects PTPRQ function is not yet known.

Although LET-805 has the structure of a cell-surface receptor protein, it has been unclear whether it interacts with specific ligands, or whether such an interaction might affect intracellular signals. Our identification of the cluster of gain-of-function mutations in the ECD as suppressors of BM mutant defects might suggest that this region of LET-805 directly interacts with one or more BM proteins. Our suppression analysis indicates that the *let-805* suppressors can suppress phenotypes due to loss of function in multiple BM proteins, but do not suppress null mutations in certain structural BM components. This pattern of suppression argues against a direct interaction mechanism, but is more consistent with bypass suppression in which the functional requirement for certain BM proteins has been relieved.

We consider two general models for how this might occur, although these are not mutually exclusive. In the first model, the gain of function mutations cause suppression by altering or enhancing interactions with specific ligand(s). Although binding partners for the LET-805 ECD are not known, candidates include BM components such as those identified as suppressors in our screen. The clustering of the LET-805 suppressors suggests that they could affect a single binding interaction. Additionally, the aggregation observed in LET-805::GFP could indicate an increased binding affinity of the mutant protein for a ligand. The second model is that the gain-of-function mutations promote BM or epidermal function more indirectly, independent of a specific ligand interaction, possibly by stabilizing a specific conformation of LET-805 that enhances its signaling activity. The mechanism of LET-805 activation or regulation is not understood but might involve lateral associations, mediated by its ECD, to form dimeric or higher-order complexes, analogous to other transmembrane receptors. Fibronectin is known interact homophilically, and its FNIII repeats have been hypothesized to be important for forming networks in the extracellular matrix (Ingham *et al.* 1997). The aggregation of LET-805::GFP might reflect analogous increased self-association into higher-order complexes. It remains to be elucidated whether or how LET-805 might signal into cells; it contains a small, highly disordered intracellular domain of unknown function.

### Mechanism of suppression by mutations in perlecan

The third major locus identified in our suppressor screen is *unc-52*/perlecan, a key conserved molecule in metazoan BMs. Perlecan is a large multidomain HSPG linked to many developmental and physiological processes (Gubbiotti *et al.* 2017). The five major domains of perlecan all likely function in protein–protein interactions (Iozzo 2005), and the missense mutations identified here could alter affinity of perlecan for specific BM proteins. *C. elegans* UNC-52 is essential for embryonic development, although it does not appear to be a generic BM component, and it remains unclear if UNC-52 is heparan sulfated. Perlecan incorporation into BMs is thought to be dependent on type IV collagen; genetic analysis has suggested that perlecan can antagonize the function of type IV collagen in the *Drosophila* BM (Pastor-Pareja and Xu 2011). Perlecan and type IV collagen also have antagonistic roles in *C. elegans* synaptic growth (Qin *et al.* 2014). These observations suggest that the suppressor mutations identified here may not all cause a gain of function, but might weakly reduce function of perlecan.

In summary, we have found that putative gain-of-function alterations in a variety of genes are capable of suppressing profound defects in BM function. These observations suggest that although BM proteins such as SPON-1 and PXN-2 are required for viability in *C. elegans*, they are not essential for BM formation. Conversely, genetic suppressor analysis suggests that other proteins, such as type IV collagen or perlecan, are essential for BM structure and cannot be bypassed, although partial loss-of-function or hypomorphic alleles of such structural BM components can be bypassed. Although the mechanistic basis of the phenotypic suppression remains to be fully explored, our results suggest unexpected levels of flexibility in the genetic requirements for BM function. It would be interesting to explore if comparable compensatory interactions might occur in the BMs of other species.

## Supplementary Material

Supplemental material is available online at www.genetics.org/lookup/suppl/doi:10.1534/genetics.118.300731/-/DC1.
