# Supplementary material for: Genetic Suppression of Basement Membrane Defects in Caenorhabditis elegans by Gain of Function in Extracellular Matrix and Cell-Matrix Attachment Genes
Source: Genetics. 2018 Feb 12;208(4):1499–512. doi: 10.1534/genetics.118.300731 (PMC5887144; doi:10.1534/genetics.118.300731)
Supplement: Supplementary file 1 [file 1499FileS1.pdf]

SUPPLEMENTAL FIGURE LEGENDS

Figure S1 Evidence for gain of function nature of suppressors. (A) RNAi of *emb-9,* which is aphenotypic in a wild type background, abrogates the suppression of both *emb-9(ju1197)* and *let-2(ju1166)* type IV collagen suppressors. (B) *let-2(ju1166)* heterozygotes are genetically recessive for suppression of *pxn-2*; only the expected 25% of progeny predicted to be homozygous are viable. (C) Heterozygous *let-805(ju1123)* C1424R is a semi-dominant suppressor of *pxn-2(tm3464)*. (D) RNAi for *let-805* causes wild type worms to become sick and completely eliminates the *let-805(ju1123)* suppression of *pxn-2(tm3464)*. (E) GFP RNAi targeting GFP in the *let-805*::GFP-3xFlag has no effect in *let-805(ju1123)::*GFP background, but abrogates the ability of *let-805(ju1123)::*GFP to suppress *pxn-2(tm3464)*. (F) Heterozygous *unc-52(ju1168)* C402Y is a semi-dominant suppressor of *pxn-2(tm3464)*.

Figure S2 (A) Alignment of 36 predicted FNIII repeats in LET-805. The original analysis of LET-805 structure suggested it contains at least 32 FNIII repeats. Reanalysis of the LET-805 extracellular domain using new tools (SMART, Pfam, ProSite) indicated an additional 4 predicted FNIII repeats as well as those previously identified. The new repeats are numbered 4, 6, 7, and 36 in this alignment. Unique sequences near the suppressor mutation clusters are underlined in red. (B) Alignment of *C. elegans* LET-805 FNIII repeats 9, 10, 14, and 15 with human sidekick-1 isoform 1 (NP_689957). Residues mutated in suppressor alleles highlighted in red. (C) Alignment of *C. elegans* LET-805 FNIII repeats 9, 10, 14, and 15 with the orthologous sequences in other nematodes (*C. briggsae*, *C. remanei*, and *P. pacificus*). Residues mutated in suppressor alleles are highlighted in yellow.

Figure S3 Predicted structures of the LET-805 FNIII repeats affected by suppressor alleles. (A) Expasy modeled electrostatic plot for LET-805 FNIII repeats 8-12, including residues R970, V1052, and R1076 (two 3-D orientations). (B) Expasy modeled electrostatic plot for LET-805 FNIII repeats 12-16, including residues P1422, C1424, R1443, G1541, E1547, A1552, G1578, R1584, and S1594 (two 3-D orientations). (C) Additional examples of structural models for the LET-805 FNIII repeats for comparison, a protein region not containing any suppressor residues was modeled.

Figure S4 *let-805* gain of function alleles do not suppress epidermal attachment defects due to loss of function in intracellular cytoskeletal components, including mutations in *vab-10a* spectraplakin, *ifb-1*/intermediate filament B1, *vab-19* Kank, and the *hecd-1* Ubiquitin Ligase.

Figure S5 LET-805::GFP localization is severely disrupted in *spon-1* and *pxn-2* loss of function embryos. The suppressor *let-805(ju1123)* restores LET-805::GFP hemidesmosome localization to wild-type-like. Confocal images of late stage mutant embryos: (A) *spon-1(ju430); let-805::GFP(ju1448)*, (B) *spon-1(ju430); let-805(ju1123)::GFP(ju1449)*, (C) *let-805::GFP(ju1448); pxn-2(ju358)*, (D) *let-805(ju1123)::GFP(ju1449); pxn-2(ju358)*, (E) *let-805(ju1123)::GFP(ju1449); pxn-2(tm3464)*.

SUPPLEMENTAL TABLES

Table S1 Strains, genotypes, and lethality quantitation

Table S2 RNAi

Table S3 Primers

Supplemental Material for Gotenstein et al.

Supplemental Table S1: Strains, Genotypes and Lethality Quantitation

Strain	Genotype	Embryonic Lethality		Larval Lethality		Adult Vab morphology		Adult wt morphology		n	Temp
CZ14391	*vab-10(ju958) I*	0%	4	0%	0	0%	2	100%	1458	1464	20C
CZ22597	*vab-10(ju1299) I*	0%	8	0%	0	0%	1	99%	1620	1629	20C
CZ22598	*vab-10(ju1300) I*	1%	11	0%	0	0%	2	99%	1688	1701	20C
CZ5772	*pxn-2(ju358) X*	24%	134	34%	194	36%	206	6%	34	568	20C
CZ11987	*vab-10(ju958) I; pxn-2(ju358) X*	2%	14	0%	2	2%	20	96%	897	933	20C
CZ13110	*vab-10(ju958) I; pxn-2(ju358) X*	3%	26	3%	30	10%	85	84%	749	890	20C
CZ14537	*vab-10(ju958) I; pxn-1(ok785) V; pxn-2(ju358) X*	3%	30	2%	29	9%	107	86%	1032	1198	20C
CZ23109	*vab-10(ju1299) I; pxn-2(ju358) X*	22%	270	9%	111	51%	618	18%	224	1223	20C
CZ24298	*vab-10(ju1300) I; pxn-2(ju358) X*	22%	195	14%	122	24%	215	41%	370	902	20C
FX03464	*pxn-2(tm3464) X*	27%	58	73%	159	0%	1	0%	0	218	20C
CZ12415	*vab-10(ju958) I; pxn-2(tm3464) X*	14%	81	41%	238	39%	225	6%	35	579	20C
	vab-10(ju958) I; pxn-1(ok785) V; pxn-2(tm3464) X	5%	14	27%	78	42%	122	26%	76	290	20C
CZ4733	*spon-1(ju430) II*	53%	245	45%	206	2%	10	0%	0	461	20C
CZ14537	*vab-10(ju958) I; spon-1(ju430) II*	39%	234	41%	246	20%	124	0%	1	605	20C
**Strain**	**Genotype**	**Embryonic Lethality**		**Larval Lethality**		**Adult Vab morphology**		**Adult wt morphology**		**n**	**Temp**
DH189	*emb-9(b189) III*	2%	8	0%	1	1%	5	97%	495	509	20C
DH246	*let-2(b246) X*	1%	10	0%	0	10%	166	89%	1491	1667	20C
CZ4322	*pxn-2(ju436) X*	11%	104	2%	21	10%	95	78%	766	986	20C
CZ18261	*emb-9(b189) III; pxn-2(ju436) X*	42%	175	18%	75	5%	20	35%	148	418	20C
CZ24028	*let-2(b246):pxn-2(ju436) X*	28%	180	17%	106	31%	199	24%	152	637	20C
RB914	*pxn-1(ok785) V*	1%	7	0%	0	0%	0	99%	1307	1314	20C
CZ18090	*emb-9(b189) III ;pxn-1(ok785) V*	7%	13	1%	1	4%	7	88%	158	179	20C
CZ24983	*pxn-1(ok785) V; let-2(b246) X*	1%	18	0%	0	0%	6	98%	1527	1551	20C
NF1101	*let-2(k193) X*	14%	22	0%	0	0%	0	86%	135	157	20C
CZ4362	*pxn-2(ju432) X*	5%	41	3%	25	19%	164	74%	653	883	20C
CZ21815	*let-2(k193):pxn-2(ju432) X*	31%	19	28%	17	20%	12	21%	13	61	20C
CZ16969	*emb-9(b189) III; pxn-2(ju358) X; Ppxn-2::PXN-2(juEx2140)*									ND	

CZ4733	*spon-1(ju430) II*	53%	245	45%	206	2%	10	0%	0	461	20C
CZ22389	*spon-1(ju430) II; emb-9(b189) III*	71%	575	25%	200	4%	33	0%	0	808	20C
CZ24150	*spon-1(ju430) II; let-2(b246) X*	85%	1078	13%	169	1%	18	0%	0	1265	20C
CZ20580	*spon-1(ju430) II; let-2(ju1166) X*	2%	11	9%	60	46%	299	43%	278	648	20C
CZ20587	*spon-1(ju430) II; let-2(ju1180) X*	23%	78	8%	26	30%	101	38%	128	333	20C
CZ22017	*spon-1(ju430) II; emb-9(ju1197) III*	33%	160	4%	20	20%	99	42%	205	484	20C
CZ16462	*pxn-2(ju430) X; let-2 gDNA(juEx3911)*	48%	58	43%	52	8%	10	0%	0	120	20C
CZ25085	*pxn-2(ju430) X; emb-9 gDNA(juEx4358)*	70%	317	17%	76	14%	62	0%	1	456	20C
CZ4734	*spon-1(e2623) II*	2%	24	0%	4	16%	213	81%	1060	1301	20C
CZ24213	*spon-1(e2623) II; emb-9(b189) III*	2%	8	0%	2	1%	5	96%	395	410	20C
CZ24212	*spon-1(e2623) II; let-2(b246) X*	1%	7	0%	0	8%	85	92%	997	1089	20C
CZ19652	*let-805(ju1123) III*	0%	1	0%	0	0%	0	100%	1052	1053	20C
CZ19894	*let-805(ju1123): emb-9(b189) III*	2%	8	0%	0	0%	1	98%	413	422	20C
CZ24028	*let-805(ju1123) III; let-2(b246) X*	0%	6	0%	0	0%	3	99%	1441	1450	20C
CZ14391	*vab-10 (ju958) I*	0%	4	0%	0	0%	2	100%	1458	1464	20C
CZ20036	*vab-10 (ju958) I; emb-9(b189) III*	0%	2	0%	2	0%	1	99%	456	461	20C
CZ5772	*pxn-2(ju358) X*	24%	134	34%	194	36%	206	6%	34	568	20C
CZ15380	*pxn-2(ju358) X; let-2 gDNA(juEx3911)*	14%	58	25%	103	45%	186	15%	63	410	20C
CZ15960	*pxn-2(ju358) X; let-2 gDNA(juEx3913)*	16%	77	27%	130	49%	233	8%	38	478	20C
CZ15959	*pxn-2(ju358) X; let-2 gDNA(juEx3914)*	16%	137	32%	272	47%	390	5%	39	838	20C
CZ15961	*pxn-2(ju358) X; emb-9 gDNA(juEx4356)*	11%	36	31%	103	4%	14	54%	182	335	20C
CZ15968	*pxn-2(ju358) X; emb-9 gDNA(juEx4358)*	1%	3	8%	28	38%	142	53%	198	371	20C
CZ16464	*pxn-2(ju358) X; emb-9 gDNA(juEx4359)*	8%	31	11%	44	40%	153	41%	158	386	20C
CZ15962	*pxn-2(ju358) X; emb-9 + let-2 gDNA(juEx4362)*	16%	14	31%	27	40%	35	13%	11	87	20C
CZ15963	*pxn-2(ju358) X; emb-9 + let-2 gDNA(juEx4363)*	22%	44	30%	60	32%	64	15%	29	197	20C
CZ16465	*pxn-2(ju358) X; emb-9 + let-2 gDNA(juEx4365)*	6%	30	23%	110	65%	308	5%	24	472	20C
FX03464	*pxn-2(tm3464) X*	27%	58	73%	159	0%	1	0%	0	218	20C
CZ22767	*emb-9(ju1197) III; pxn-2(tm3464) X*	8%	62	32%	241	9%	70	51%	391	764	20C
CZ24634	*let-2(ju1166):pxn-2(tm3464) X*	8%	44	15%	76	52%	268	25%	132	520	20C
CZ21496	*let-2(ju1180):pxn-2(tm3464) X*	16%	46	18%	54	61%	182	5%	14	296	20C
CZ11147 x CZ24634	*let-805(ju1166)/+ III; pxn-2(tm3464) X*	36%	38	42%	44	22%	23	0%	0	105	20C
		Emb/Let		Sterile		wt		Males		Total	
CH1179	*unc-36(e251) emb-9(g23cg46) III / qC1 III*	15%	130	24%	205	53%	453	0%	0	853	20C

CZ25327	*emb-9(ju1197) III males x*<br>*unc-36(e251) emb-9(g23cg46) III / qC1 III herm*	3%	26	2%	20	57%	469	37%	307	822	20C
**Strain**	**Genotype**	**Embryonic Lethality**		**Larval Lethality**		**Adult Vab morphology**		**Adult wt morphology**		**n**	**Temp**
CZ4733	*spon-1(ju430) II*	28%	140	71%	352	0%	1	0%	0	493	25C
CZ20594	*spon-1(ju430) II; let-805(ju1184) III*	4%	32	0%	3	3%	26	92%	710	771	25C
CZ23913	*spon-1(ju430) II; let-805(ju1386) III*	21%	149	4%	27	15%	110	60%	438	724	25C
CZ20579	*spon-1(ju430) II; let-805(ju1165) III*	8%	27	1%	2	39%	126	53%	172	327	25C
CZ20589	*spon-1(ju430) II; let-805(ju1177) III*	30%	220	2%	17	16%	119	52%	382	738	25C
CZ19892	*spon-1(ju430) II; let-805(ju1123) III*	22%	98	3%	13	33%	147	42%	184	442	25C
CZ20585	*spon-1(ju430) II; let-805(ju1173) III*	6%	26	4%	16	57%	259	34%	152	453	25C
CZ19891	*spon-1(ju430) II; let-805(ju1122) III*	36%	108	2%	7	32%	95	30%	89	299	25C
CZ20590	*spon-1(ju430) II; let-805(ju1175) III*	38%	294	7%	53	28%	216	27%	211	774	25C
CZ20599	*spon-1(ju430) II; let-805(ju1198) III*	18%	151	20%	165	35%	299	27%	230	845	25C
CZ20581	*spon-1(ju430) II; let-805(ju1167) III*	23%	104	10%	46	44%	196	22%	99	445	25C
CZ20584	*spon-1(ju430) II; let-805(ju1170) III*	27%	124	10%	46	42%	192	22%	100	462	25C
CZ20597	*spon-1(ju430) II; let-805(ju1187) III*	10%	73	19%	145	64%	480	7%	53	751	25C
CZ22015	*spon-1(ju430) II; let-805(ju1190) III*	14%	68	33%	165	46%	230	6%	32	495	25C
**Strain**	**Genotype**	**Embryonic Lethality**		**Larval Lethality**		**Adult Vab morphology**		**Adult wt morphology**		**n**	**Temp**
FX03464	*pxn-2(tm3464) X*	27%	58	73%	159	0%	1	0%	0	218	20C
CZ18265	*let-805(ju1123) III; pxn-2(tm3464) X*	6%	31	1%	7	32%	177	61%	337	552	20C
CZ23912	*let-805(ju1386) III; pxn-2(tm3464) X*	1%	7	0%	2	46%	252	52%	281	542	20C
CZ18264	*let-805(ju1122) III; pxn-2(tm3464) X*	5%	25	1%	5	46%	230	48%	242	502	20C
CZ20991	*let-805(ju1165) III; pxn-2(tm3464) X*	3%	26	1%	11	61%	463	35%	265	765	20C
CZ21498	*let-805(ju1184) III; pxn-2(tm3464) X*	27%	172	6%	38	53%	341	14%	93	644	20C
CZ20988	*let-805(ju1170) III; pxn-2(tm3464) X*	14%	101	22%	155	50%	351	14%	100	707	20C
CZ22296	*let-805(ju1187) III; pxn-2(tm3464) X*	2%	5	1%	2	90%	252	8%	21	280	20C
CZ20990	*let-805(ju1173) III; pxn-2(tm3464) X*	22%	101	39%	175	35%	157	4%	19	452	20C
CZ11147<br>x<br>CZ18265	*let-805(ju1123)/+ III; pxn-2(tm3464) X*	23%	86	16%	59	54%	203	7%	27	375	20C
**Strain**	**Genotype**	**Embryonic Lethality**		**Larval Lethality**		**Adult Vab morphology**		**Adult wt morphology**		**n**	**Temp**

N2	-	0%	1	0%	0	0%	0	100%	1249	1250	20C
CZ19652	*let-805(ju1123) III*	0%	1	0%	0	0%	0	100%	1052	1053	20C
CZ5772	*pxn-2(ju358) X*	24%	134	34%	194	36%	206	6%	34	568	20C
CZ20030	*let-805(ju1123) III; pxn-2(ju358) X*	1%	6	0%	0	0%	3	99%	665	674	20C
FX03464	*pxn-2(tm3464) X*	27%	58	73%	159	0%	1	0%		218	20C
CZ19654	*let-805(ju1123) III; pxn-2(tm3464) X*	6%	31	1%	7	32%	177	61%	337	552	20C
RB914	*pxn-1(ok785) V*	1%	7	0%	0	0%	0	99%	1307	1314	20C
CZ19896	*let-805(ju1123) III; pxn-1(ok785) V*	0%	1	0%	0	0%	2	100%	1477	1480	20C
CZ4733	*spon-1(ju430ts) II*	53%	245	45%	206	2%	10	0%	0	461	20C
CZ19892	*spon-1(ju430ts) II; let-805(ju1123) III*	2%	12	0%	0	2%	15	96%	735	762	20C
CZ6767	*spon-1(ju402) II*	51%	92	48%	86	1%	2	0%	0	180	20C
CZ20368	*spon-1(ju402) II; let-805(ju1123) III*	2%	18	0%	2	29%	328	70%	799	1147	20C
DH189	*emb-9(b189ts) III*	81%	257	18%	56	1%	3	0%	1	317	22.5C
CZ19894	*let-805(ju1123):emb-9(b189ts) III*	36%	65	8%	14	34%	60	22%	40	179	22.5C
NF1973	*emb-9(tk75) III*	78%	350	1%	4	0%	0	21%	94	448	20C
CZ22594	*let-805(ju1123):emb-9(tk75) III*	66%	445	2%	12	2%	12	31%	208	677	20C
DH246	*let-2(b246) X*	80%	354	3%	15	2%	8	15%	64	441	22.5C
CZ24028	*let-805(ju1123) III; let-2(b246) X*	2%	26	0%	0	1%	9	97%	1277	1312	22.5C
NF1101	*let-2(k193) X*	14%	22	0%	0	0%	0	86%	135	157	20C
CZ23862	*let-805(ju1123) III; let-2(k193) X*	8%	6	0%	0	0%	0	92%	73	79	20C
CZ23138	*mec-7-GFP(muIs32) II; epi-1(gm121) IV*	81%	485	8%	51	4%	27	6%	39	602	20C
CZ23793	*mec-7-GFP(muIs32) II; let-805(ju1123) III; epi-1(gm121) IV*	2%	20	0%	0	16%	185	83%	977	1182	20C
CB698	*vab-10(e698) I*	1%	13	0%	1	53%	586	46%	503	1103	20C
CZ20365	*vab-10(e698) I; let-805 (ju1123) III*	1%	11	0%	4	53%	452	45%	389	856	20C
CZ4380	*ifb-1(ju71) II*	25%	287	0%	0	67%	779	9%	104	1170	20C
CZ20363	*ifb-1(ju71) II; let-805 (ju1123) III*	29%	245	0%	2	63%	526	7%	60	833	20C
CZ401	*vab-19(e1036) II*	35%	436	11%	141	22%	271	32%	391	1239	20C
CZ20077	*vab-19(e1036) II; let-805 (ju1123) III*	32%	412	16%	208	21%	275	31%	401	1296	20C
CZ3743	*vab-19(ju406) II*	88%	685	12%	91	0%	0	0%	0	776	20C
CZ20995	*vab-19(ju406) II; let-805 (ju1123) III*	88%	654	12%	87	0%	0	0%	0	741	20C
RB1319	*hecd-1(ok1437) IV*	6%	53	0%	0	1%	7	93%	801	861	20C
CZ22914	*let-805(ju1123) III; hecd-1(ok1437) IV*	6%	65	0%	3	0%	1	94%	1069	1138	20C

**Strain**	**Genotype**
CZ23945	*let-805::GFP::3xFlag(ju1448) III*

CZ23946	*let-805(ju1123) let-805::GFP::3xFlag(ju1449) III*
CZ24114	*let-805(ju1123) III let-805::GFP::3xFlag(ju1449) III ; pxn-2(tm3464) X*
CZ24278	*let-805::GFP::3xFlag(ju1448) III ; pxn-2(ju358) X*
CZ24279	*let-805(ju1123) III let-805::GFP::3xFlag(ju1449) III ; pxn-2(ju358) X*
CZ24280	*spon-1(ju430ts) II ; let-805::GFP::3xFlag(ju1448) III*
CZ24281	*spon-1(ju430ts) II ; let-805(ju1123) III let-805::GFP::3xFlag(ju1449) III*
CZ26312	*let-805(ju1165) let-805::GFP::3xFlag(ju1628) III*
CZ26314	*let-805(ju1184) let-805::GFP::3xFlag(ju1630) III*

**Strain**	**Genotype**	**Embryonic Lethality**		**Larval Lethality**		**Adult Vab morphology**		**Adult wt morphology**		**n**	**Temp**
FX03464	*pxn-2(tm3464) X*	27%	58	73%	159	0%	1	0%	0	218	20C
CZ21257	*unc-52(ju1168) II; pxn-2(tm3464) X*	2%	25	0%	3	20%	228	78%	894	1150	20C
CZ24047	*unc-52(ju1172) II; pxn-2(tm3464) X*	3%	33	8%	76	65%	624	23%	220	953	20C
CZ21494	*unc-52(ju1174) II; pxn-2(tm3464) X*	17%	145	2%	19	53%	458	28%	243	865	20C
CZ11147 x CZ21257	*let-805(ju1168)/+ III; pxn-2(tm3464) X*	15%	74	29%	142	31%	152	25%	124	492	20C
CZ4733	*spon-1(ju430) II*	28%	140	71%	352	0%	1	0%	0	493	25C
CZ20582	*unc-52(ju1168): spon-1(ju430) II*	4%	25	2%	12	24%	150	70%	430	617	25C
CZ23560	*unc-52(ju1172): spon-1(ju430) II*	47%	217	4%	18	20%	91	29%	135	461	25C
CZ20588	*unc-52(ju1174): spon-1(ju430) II*	33%	194	3%	18	31%	183	32%	186	581	25C
DH189	*emb-9(b189ts) III*	81%	257	18%	56	1%	3	0%	1	317	22.5C
CZ25289	*unc-52(ju1168) II; emb-9(b189ts) III*	55%	230	10%	44	23%	95	12%	52	421	22.5C
CZ25287	*unc-52(ju1172) II; emb-9(b189ts) III*	26%	76	13%	40	40%	118	21%	63	297	22.5C
CZ25288	*unc-52(ju1174) II; emb-9(b189ts) III*	31%	133	7%	32	34%	148	27%	118	431	22.5C
		Embryonic Lethality		Larval Lethality		Unc		Non-Unc		n	Temp
CB444	*unc-52(e444) II*	1%	1	0%	0	99%	125	0%	0	126	20C
CZ23388	*unc-52(e444) II; let-805(ju1123) III*	0%	0	0%	0	100%	161	0%	0	161	20C
CZ23666	*unc-52(e444) II; pxn-2(ju436) X*									ND	

Whole Genome Sequenced strains

Strain	Genotype	Notes
CZ13800	*pxn-2(ju358) X*	WGS version of CZ5772
CZ13801	*vab-10(ju958) I ; pxn-2(ju358) X*	1x outcrossed to N2
CZ13802	*vab-10(ju958) I ; pxn-2(tm3464) X*	WGS version of CZ12415
CZ18264	*let-805(ju1122) III ; pxn-2(tm3464) X*	2x outcrossed
CZ18265	*let-805(ju1123) III ; pxn-2(tm3464) X*	2x outcrossed
CZ20579	*spon-1(ju430ts) II ; let-805(ju1165) III*	1x outcrossed
CZ20580	*spon-1(ju430ts) II ; let-2(ju1166) X*	1x outcrossed
CZ20581	*spon-1(ju430ts) II ; let-805(ju1167) III*	1x outcrossed
CZ20988	*let-805(ju1170) III ; pxn-2(tm3464) X*	
CZ20990	*let-805(ju1173) III ; pxn-2(tm3464) X*	
CZ21257	*unc-52(ju1168) II ; pxn-2(tm3464) X*	
CZ22017	*spon-1(ju430ts) II ; emb-9(ju1197) III*	1x outcrossed
CZ21494	*unc-52(ju1174) II ; pxn-2(tm3464) X*	
CZ21496	*pxn-2(tm3464) X : let-2(ju1180) X*	
CZ22296	*let-805(ju1187) III ; pxn-2(tm3464) X*	
CZ22297	*unc-52(ju1188) II ; pxn-2(tm3464) X*	
CZ23560	*spon-1(ju430ts) II : unc-52(ju1172) II*	1x outcrossed

Supplemental Material for Gotenstein et al.

Supplemental Table S2: RNAi

Worm Strain	Genotype	RNAi	Embryonic Lethality		Larval Lethality		Vab Adults		WT Adults		n
N2	Wild-type	Empty vector	0%	0	0%	0	0%	0	100%	473	719
		emb-9	0%	0	0%	0	0%	0	100%	394	616
CZ11147	*pxn-2(tm3464) X*	Empty vector	59%	102	40%	68	1%	2	0%	0	216
		emb-9	39%	56	53%	76	8%	11	0%	0	158
CZ22767	*emb-9(ju1197) III; pxn-2(tm3464) X*	Empty vector	2%	2	6%	6	33%	35	59%	62	765
		emb-9	15%	13	7%	6	33%	28	44%	37	678
CZ24634	*emb-9(ju1197) III; pxn-2(tm3464) X*	Empty vector	0%	0	1%	1	24%	18	75%	56	203
		emb-9	11%	6	11%	6	24%	13	55%	30	152

Worm Strain	Genotype	RNAi	Embryonic Lethality		Larval Lethality		Vab Adults		WT Adults		n
N2	Wild-type	Empty vector	0%	0	0%	0	0%	0	100%	473	719
		let-805	0%	1	1%	6	98%	556	1%	7	616
CZ11147	*pxn-2(tm3464) X*	Empty vector	59%	102	40%	68	1%	2	0%	0	216
		let-805	90%	126	7%	10	3%	4	0%	0	158
CZ19892	*let-805(ju1123) III ; pxn-2(tm3464) X*	Empty vector	0%	0	0%	1	21%	45	79%	169	765
		let-805	78%	131	22%	36	0%	0	0%	0	678

Worm Strain	Genotype	RNAi	Embryonic Lethality		Larval Lethality		Vab Adults		WT Adults		n
CZ23946	*let-805(ju1123) let-805::GFP::3xFlag(ju1449) III*	Empty vector	0%	0	0%	0	0%	0	100%	677	719
		GFP	1%	3	0%	0	0%	0	99%	558	616
CZ11147	*pxn-2(tm3464) X*	Empty vector	59%	102	40%	68	1%	2	0%	0	216
		GFP	63%	109	35%	61	2%	3	0%	0	158
CZ19892	*let-805(ju1123) III ; pxn-2(tm3464) X*	Empty vector	0%	0	0%	1	21%	45	79%	169	765
		GFP	0%	0	0%	0	17%	15	83%	71	678
CZ24114	*let-805(ju1123) III let-805::GFP::3xFlag(ju1449) III ; pxn-2(tm3464) X*	Empty vector	0%	0	0%	0	11%	14	89%	114	203
		GFP	2%	3	4%	7	41%	70	53%	90	152

Supplemental Table S3: Primers

Primer #	Name	Sequence	Purpose
AC1744	ju358 D1	GAAAAGTTACGACGGCAATC	genotyping tm3464
AC1752	PXN-2 P4	GCAGTGAGTGTGAGATCGTG	
AC2047	PXN-2 P13	GAAAGTTGCGAAGATCACTGC	
AC1642	ju430 d1	GTGGATGATGGTGGTGAAAGAGT	genotyping ju430
AC1643	ju430 u1	TCGCCCCTGATACCCATTTAC	
AC1736	emb-9 b189 For	ACAAAACTTTCAGGGTTTCCC	genotyping b189 and ju1197
AC1737	emb-9 b189 Rev	CCCTTTTCTCCTTTCACTCCTG	
AC3781	let-2 For ju1166	CCCAGGACAAAGCATCCCAG	genotyping ju1166 and ju1180
AC3782	let-2 Rev ju1166	TCCTGGTCTAAGGGTTAGCTG	
AC3623	vab-10 ju958 us	GCATCTCACCACTCTGGCAG	genotyping ju958, ju1299, and ju1300
AC3624	vab-10 ju958 ds	TGATTGACACGCTGGGTTGG	
AC3299	let-805 For	TTGCGACAACAACCAGGAGG	genotyping ju1123, ju1122, ju1165, ju1167, ju1175, ju1177, ju1184, ju1187, ju1198
AC3300	let-805 Rev	CCGCAGATTCGGTGACCTTAG	
AC3661	let-805 p11 For	TTTTCACAGCTCGAATGTCC	genotyping ju1170, ju1173, ju1190
AC3674	let-805 p28 Rev	TTGCACTGGGATTGTGTTTG	
AC3763	unc-52 ju1174 For	ACCACTTCCATCTTCTGCTC	genotyping ju1174
AC3764	unc-52 ju1174 Rev	ATAAGGCTCAGGATGGGTTC	
AC3765	unc-52 ju1168 For	GACTGCATCAAACCGTTGAG	genotyping *ju1168*
AC3766	unc-52 ju1168 Rev	CTTACACAAGCGGCGTCCTG	
AC3997	unc-52 ju1172 For	ACTTCAATCGGAGCCAGTGC	genotyping *ju1172*
AC3998	unc-52 ju1172 Rev	CTGTTAGTGGTTGGGTCCGTG	
AC3961	let-805 C-term 5' arm For	ACGTTGTAAAACGACGGCCAGTCGCCGGCACTCTGCAACCCAA CGTAATTC	*let-805* C-terminal GFP Crispr knock-in
AC3962	let-805 C-term 5' arm Rev	CATCGATGCTCCTGAGGCTCCCGATGCTCCACGGGATGAGTGCT CTTCTTTG	
AC3963	let-805 C-term 3' arm For	CGTGATTACAAGGATGACGATGACAAGAGATAATCTCTGGAAA TTTTCAACC	
AC3964	let-805 C-term 3' arm Rev	GGAAACAGCTATGACCATGTTATCGATTTCTCTGAGCACTGAAC CCACTG	
AC3965	let-805 C-term sgRNA F	GCACTCATCCCGTTAATCTCGTTTTAGAGCTAGAAATAGCAAGT TAAAATAAG	
AC3966	let-805 C-term sgRNA R	GAGATTAACGGGATGAGTGCCAAGACATCTCGCAATAGGAG	
AC3858	let-805 ju1123 sgRNA F	CCATTGGTCTGAAGGCATGGGTTTTAGAGCTAGAAATAGCAAG	*ju1123* C1424R (T>C) Crispr generated SNP

		TTAAAATAAG	
AC3859	let-805 ju1123 sgRNA R	CCATGCCTTCAGACCAATGGCAAGACATCTCGCAATAGGAG	
AC3790	ju1123 crispr oligo	tacctccgaacagaggacattcgtccaactgatgttagtatttcctggcaggcaccaccaCgccttcagac caatggagaaatcaccgagtacgagtacgaggtaactgctggagatcgta	
AC3724	vab-10 ju958 sgRNA F	TCAACGTGACGCTATCATGAGTTTTAGAGCTAGAAATAGCAAG TTAAAATAAG	attempt to knock in *ju958* D941N (G>A, in red) SNP, instead generating deletions *ju1299* and *ju1300*
AC3725	vab-10 ju958 sgRNA R	TCATGATAGCGTCACGTTGACAAGACATCTCGCAATAGGAG	
AC3746	ju958 crispr oligo	aggatgggatctcgacacgttcaactcgattgaccctgatcaacgtgacgctatcatgaaggcactgaac gatAatgctaacaaactgctcagcgagctcgatccaaatgatccattggcg	

Figure S1

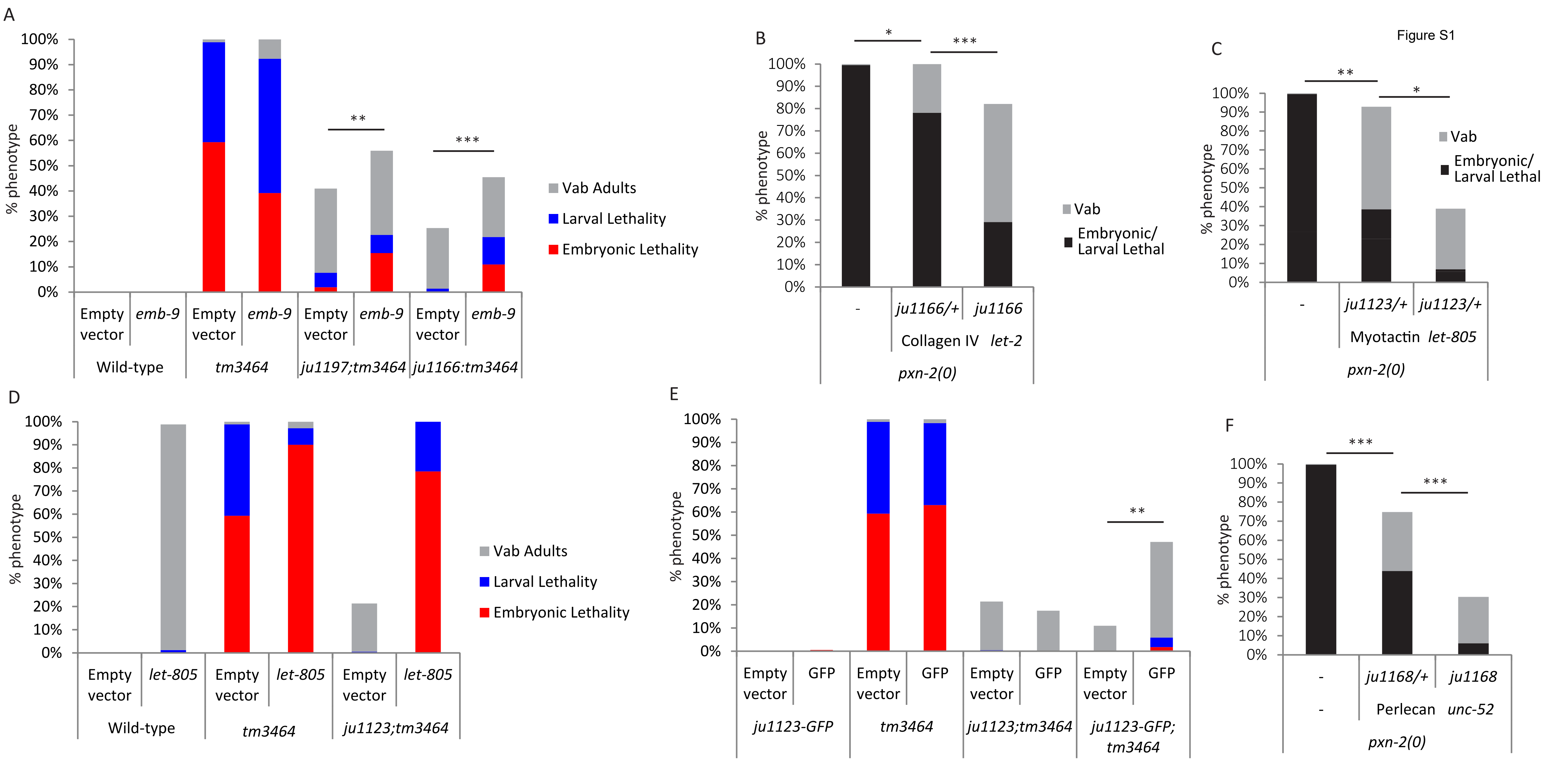
A
% phenotype
100%
90%
80%
70%
60%
50%
40%
30%
20%
10%
0%
**
***
Empty vector
emb-9
Wild-type
tm3464
ju1197;tm3464
ju1166:tm3464
Vab Adults
Larval Lethality
Embryonic Lethality
B
% phenotype
*
***
-
ju1166/+
ju1166
Collagen IV let-2
pxn-2(0)
Vab
Embryonic/
Larval Lethal
C
% phenotype
**
*
-
ju1123/+
Myotactin let-805
pxn-2(0)
Vab
Embryonic/
Larval Lethal
D
% phenotype
Empty vector
let-805
Wild-type
tm3464
ju1123;tm3464
Vab Adults
Larval Lethality
Embryonic Lethality
E
% phenotype
**
Empty vector
GFP
ju1123-GFP
tm3464
ju1123;tm3464
ju1123-GFP;
tm3464
F
% phenotype
***
***
-
ju1168/+
ju1168
Perlecan unc-52
pxn-2(0)

Figure S2

A

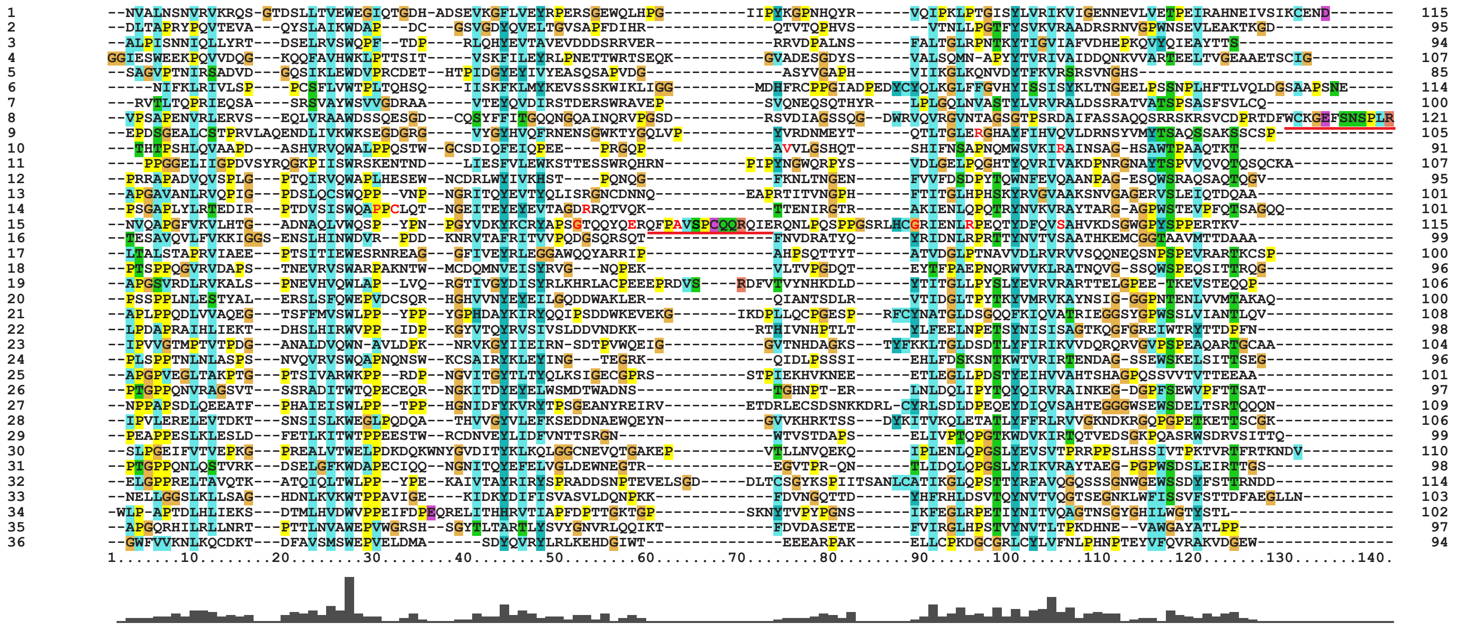

B

```
LET-805 Ce   EPDSGEALCSTPRVLAQENDLIVKWKS--EGDGRG-VYGYHVQFRNENSGWKTYGQLVPYVRDNMEYTQTLTGLERGHAYFIHVQVLDRNSYVMYTSAQSSAKSSCSPP 1003
 (FN3 9)     E  S EA+ ST          +++ W S  E D  G + GY + FR ++    +    +V   R N    +  L GL +    Y + V    R    + ++     ++     P
Sidekick Hs  ENVSAEAVSST--------QILLTWTSVPEQDQNGLILGYKILFRAKDLDPEPRSHIV---RGNHTQSALLAGLRKFVLYELQVLAFTRIGNGVPSTPLILERTKDDAP 1378

LET-805 Ce   THTPSHLQVAAPDASHVRVQWALPPQSTWGCSDIQFEIQPEEPRGQPAVVLGSHQTSHIFNSAPNQMWSVKI-------RAINSAGHSAWTPAAQTKT 1096
 (FN3 10)      P  L       + VR+ W                  PEEP G   ++LG      + +S+P+    +V++         A + A  SA+       KT
Sidekick Hs  -GPPVRLVFPEVRLTSVRIVWQ----------------PPEEPNG---IILGYQIAYRLASSSPHTFTTVEVGATVRQFTATDLAPESAYIFRLSAKT 1458

LET-805 Ce   PS--GAPLYLRTEDIRPTDVSISWQAPPCLQTNGEITEY--EYEVTA-GDRRQ--TVQKTTENIRGTRAKIENLQPQTRYNVKVRAYTARGAGPWS-TEVPFQTSAGQQ 1498
 (FN3 14)    P   GAP  L   +I P   ++ ++  P        I+ +  E +V A GD  +   T+ +              +I NL P T Y  +++      G  P+S +    QT
Sidekick Hs  PDLPGAPSNLVISNISPRSATLQFR--PGYDGKTSISRWIVEGQVGAIGDEEEWVTLYEEENEPDAQMLEIPNLTPYTHYRFRMKQVNIVGPSPYSPSSRVIQTLQAPP 1173

LET-805 Ce   NVQAPGFVKVLHTGADNAQLVWQSPYPNPGYVDK-----YKCRYAPSGTQQYQ---------ERQF----PAVSPCQQRQIERQNLPQSPPGSRLHCGRI---------ENLR---------------PEQTYD-----FQVSAHVKDSGWGPYSPPERTKV 1613
 (FN3 15)    +V AP  V V       + +L W  P P+  Y            Y+ +Y  S  Q              ER+F               + Q++  N    + P S +  GR          EN+                PEQ  +      +++    KD    P S    R
Sidekick Hs  DV-APTSVTVRTASETSLRLRW-VPLPDSQYNGNPESVGYRIKYWRSDLQSSAVAQVVSDRLEREFTIEELEEWMEYELQMQAFNAVGAGPWSEVVRGRTRESVPSAAPENVSAEAVSSTQILLTWTSVPEQDQNGLILGYKILFRAKDLDPEPRSHIVRGNH 1278
```

C

```
C.briggsae-LET-805       ATDSGEGAVNRVWSLVSTPRVLAQDNDLIVKWKSEGEGRGVYGYHVQFRSENSGWKTYGQLVPYVGDNMEYTQTLTGLERGNAYFIHVQVLDRNSYVMYTSAQSSAKSSCSPP 1010
C.remanei-LET-805        ETDSGE-------ALVSTPRVLAQDNDLIVKWKSEGEGRGVYGYHVQFRSENSGWKTYGQLVPYVGDNMEYTQTLTGLERGNAYFIHVQVLDRNSYVMYTSAQSNAKSSCSKP 1018
LET-805_A_C.elegans      EPDSGE-------ALCSTPRVLAQENDLIVKWKSEGDGRGVYGYHVQFRNENSGWKTYGQLVPYVRDNMEYTQTLTGLERGHAYFIHVQVLDRNSYVMYTSAQSSAKSSCSPP 1003
P.pacificus-LET-805      RVQSSCGGAVRACPLCSTPRVSARNGDLVVEWRSEGDGRGVHGYRIQYRTDQSGWNPYGQLVPYVGDEKEYSQTLTGLQRGHNYYMHIQVLDRNSYVMYTSPEASATSACSAP 829
                          :*.        .* ***** *::.**:*:*:***:****:**::*:*.::***:.******** *: **:******:**: *::*:*************.::.*.*:** *

C.briggsae-LET-805       THTPSHLQVAAPDASHVRVSWALPPQSTWGCSDIQFEIQPEEPRGQP--AVVLGSHQTSHIFNSAPNQMWSVKIRAINSAGHSAWTPASQAKT 1101
C.remanei-LET-805        THTPSHLQVAAPDASHVRVSWALPPQSTWGCSDIQFEIQPEEPRGQP--AVVLGSHQTSHIFNSAPNQLWSVKIRAINSAGHSAWTPASQAKT 1109
LET-805_A_C.elegans      THTPSHLQVAAPDASHVRVQWALPPQSTWGCSDIQFEIQPEEPRGQP--AVVLGSHQTSHIFNSAPNQMWSVKIRAINSAGHSAWTPAAQTKT 1094
P.pacificus-LET-805      TQAPSHIAVEAPDASHVRVTWALPPQSGWGCADITFEIQVEEPRGLHPSPQRVAGGSTSHVFVAHPGQQWTIRMRTVNSAGHSAWTAPATVKT 922
                         *::***: * ********* ******* ***:** **** *****    .  :.. .***:* : *.* *::::*::*********..: .**

C.briggsae-LET-805       PGGAPLYLRTEEIRPTDVSISWQAPPCLQTNGEITEYEYEVTAGDRRQTVQKTTENIRG-TRARIENLQPQTRYNVKVRAYTARGPGPWSTEVPFQTSAGQQ 1505
C.remanei-LET-805        PSGAPLYLRTDDIRPTDVSISWQAPPCLQTNGEITEYEYEVTAGDRRQTVQKTTENIRG-TRARIENLQPQTRYNVKVRAYTARGPGPWSTEVPFQTSAGQQ 1513
LET-805_A_C.elegans      PSGAPLYLRTEDIRPTDVSISWQAPPCLQTNGEITEYEYEVTAGDRRQTVQKTTENIRG-TRAKIENLQPQTRYNVKVRAYTARGAGPWSTEVPFQTSAGQQ 1498
P.pacificus-LET-805      PTAAPTYTRIEETTESEAAVSWQAPPCLQTHGEITEYEFEAVPLDKR--YPPNTNTVRGSTRTRITGLLPGTDYNLRVRAFTTKGPGPWSHPTRFQTAAAAA 1324
                         * .** * * ::   ::.::*********:*******:*... *:*     .*:.:** **::* .* * * **::***:*::*.****  . ***:*.

C.briggsae-LET-805       NVQAPGFVKVLHTGANNAQLVWQSPYP-NPGYVDKYKCRYAPTGTQQFQERQFPAVSPCQQRQIERQNLPQSPPGARLHCGRIENLKPEQTYDFQVSAHVKDSGWGPYSPPERTKV 1620
C.remanei-LET-805        NVQAPGFVKVLHTGADNAQLVWQSPYP-NPGYVDKYKCRYAPTGTQQYQERQFPAVSPCQQRQIERQQLPQSPPGARLHCGRIENLKPEQTYDFQVSAHVKDSGWGPYSPPERTLV 1628
LET-805_A_C.elegans      NVQAPGFVKVLHTGADNAQLVWQSPYP-NPGYVDKYKCRYAPSGTQQYQERQFPAVSPCQQRQIERQNLPQSPPGSRLHCGRIENLRPEQTYDFQVSAHVKDSGWGPYSPPERTKV 1613
P.pacificus-LET-805      SSVPP-LVKVVGTGADSAHLLWHSPPSGQPDQYNKFKCRYGITGQQQRQEQVFPAQSPCSQELIRRQQVPPPPPGSRMHCGRIDRLQQNQTYDFQVSACQRNNKCSPYSPPERSRI 1439
                         .  .* :***: ***:.*:*:*:** . :*.  :*:****. :* ** **: *** ***.*. *.**::* .***:*:*****:.*: :*********  ::.  .*******: :
```

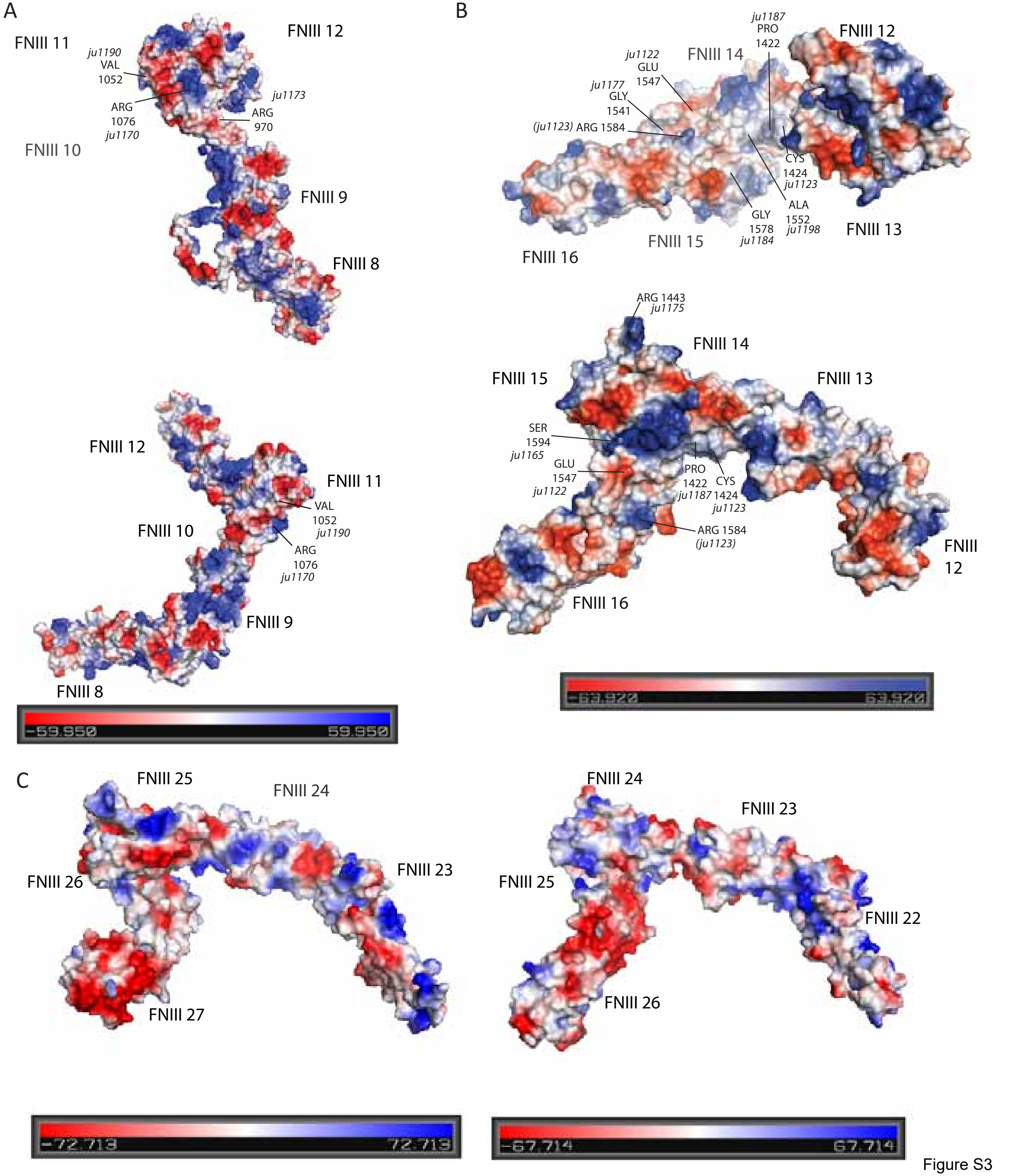

A
FNIII 11
FNIII 12
ju1190
VAL
1052
ARG
1076
ju1170
ju1173
ARG
970
FNIII 10
FNIII 9
FNIII 8
-59.950
59.950
B
ju1187
PRO
1422
FNIII 12
ju1122
GLU
1547
FNIII 14
ju1177
GLY
1541
(ju1123) ARG 1584
CYS
1424
ju1123
ALA
1552
ju1198
GLY
1578
ju1184
FNIII 13
FNIII 15
FNIII 16
ARG 1443
ju1175
SER
1594
ju1165
-63.920
63.920
C
FNIII 25
FNIII 24
FNIII 26
FNIII 23
FNIII 27
FNIII 22
-72.713
72.713
-67.714
67.714

Figure S3

Figure S4

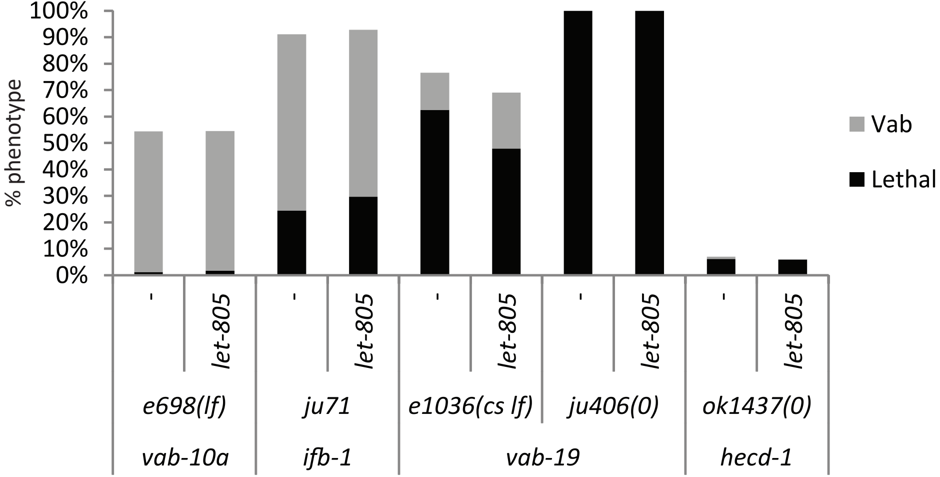

100%
90%
80%
70%
60%
50%
40%
30%
20%
10%
0%
% phenotype
Vab
Lethal
-
let-805
-
let-805
-
let-805
-
let-805
-
let-805
e698(lf)
ju71
e1036(cs lf)
ju406(0)
ok1437(0)
vab-10a
ifb-1
vab-19
hecd-1

Figure S5

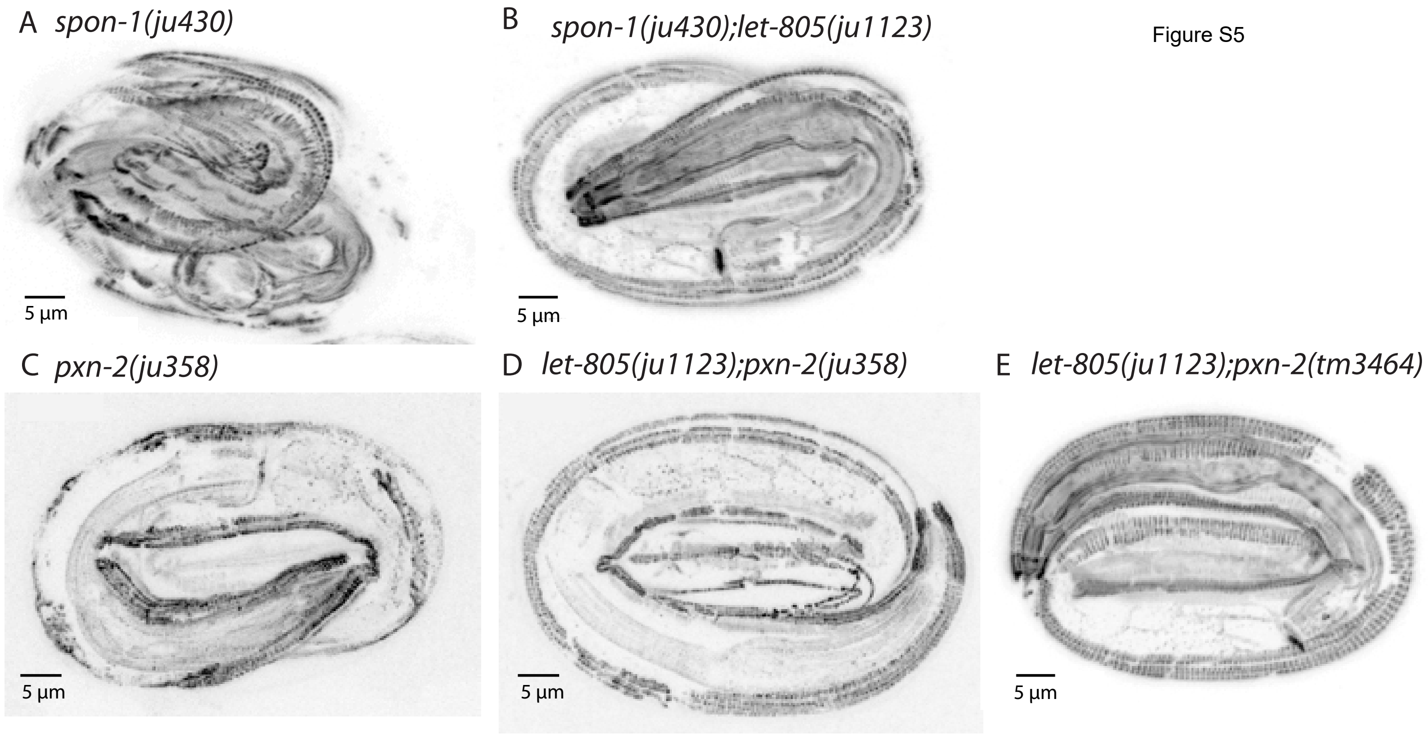
A spon-1(ju430)
B spon-1(ju430);let-805(ju1123)
C pxn-2(ju358)
D let-805(ju1123);pxn-2(ju358)
E let-805(ju1123);pxn-2(tm3464)
5 µm
5 µm
5 µm
5 µm
5 µm
